# Development of Covalently Functionalized Alginate–Pyrrole and Polypyrrole–Alginate Nanocomposites as 3D Printable Electroconductive Bioinks

**DOI:** 10.3390/ma18133120

**Published:** 2025-07-01

**Authors:** Abraham Abbey Paul, Olga Kryukov, Anil Kumar Bandela, Hamody Muadi, Nurit Ashkenasy, Smadar Cohen, Robert S. Marks

**Affiliations:** 1 Avram and Stella Goldstein-Goren Department of Biotechnology Engineering, Ben-Gurion University of the Negev, Beer Sheva 84105, Israel; paulab@post.bgu.ac.il (A.A.P.); kryukov@bgu.ac.il (O.K.); scohen@bgu.ac.il (S.C.); 2 Department of Chemistry, Ben Gurion University of Negev Beer, Beer Sheva 84105, Israel; abande7@emory.edu; 3 Department of Chemistry, Emory University, Atlanta, GA 30322, USA; 4 Department of Materials Engineering, Ben-Gurion University of the Negev, Beer Sheva 84105, Israel; muadi@post.bgu.ac.il (H.M.); nurita@bgu.ac.il (N.A.); 5 The Ilse Katz Center for Nanoscale Science and Technology, Ben-Gurion University of the Negev, Beer Sheva 84105, Israel; 6 Regenerative Medicine and Stem Cell (RMSC) Research Center, Ben-Gurion University of the Negev, Beer Sheva 84105, Israel

**Keywords:** alginate–pyrrole, 3D bioprinting, polypyrrole, conductive hydrogel, bioink, electrical conductivity, rheological properties

## Abstract

Electrically conductive hydrogels are gaining attention owing to their applications in biosensing, cellular interfaces, and tissue engineering. However, conventional hydrogels often lack adequate electrical conductivities. Here, we present two novel conductive alginate-based hydrogels designed for extrusion-based 3D bioprinting: (i) covalently synthesized alginate–polypyrrole (alginate–PPy) via EDC/NHS-mediated conjugation with 3-aminopropyl pyrrole, and (ii) nanoparticle-reinforced alginate blended with polypyrrole nanoparticles (alginate@PPy-NP). Both systems exhibited shear-thinning behavior, tunable viscoelasticity, and excellent printability. Alginate@PPy-NP demonstrated superior compressive strength and shape fidelity, whereas alginate–PPy showed enhanced elastic moduli (G′/G″), reflecting a more uniform gel network. Electrical conductivity increased with increasing pyrrole content in both formulations. Optimization of the composition and printing conditions enabled the fabrication of fibroblast-laden constructs with high structural integrity. This work highlights the potential of alginate–polypyrrole hydrogels as customizable, conductive bioinks for 3D bioprinting in regenerative medicine.

## 1. Introduction

Three-dimensional (3D) (bio)printing is a cutting-edge technology for the precise deposition of (bio)materials onto specified locations with a high resolution. This process involves fabricating objects layer by layer using a printer head, nozzle, or similar technology [1]. The convergence of additive manufacturing with biomaterials has introduced new paradigms in the field of biotechnology. The development of advanced printing materials and 3D printing technologies capable of fabricating complex scaffold geometries has facilitated the integration of sensing elements, thereby opening new avenues for biosensor design and functionality [2,3]. Three-dimensional bioprinting now extends to critical applications, including embedding active biomolecular recognition elements into 3D printed objects for (bio)sensing [2,3,4,5], applications in tissue engineering [6], wound healing [6], and the production of various medical implants and models [7]. The major challenges commonly encountered during printing processes with bio-based hydrogels are structural collapse and loss of shape fidelity, depending on the viscoelastic properties of the printing material [8]. Rapidly solidifying “inks” are desirable for printing to prevent the deformation of the printed structure. An ideal hydrogel ink should flow through a nozzle during printing and retain its shape after printing and curing, which requires custom materials with tailored properties to be formulated for each application. Therefore, several hydrogels with varying viscosities have been adapted for 3D printing via microextrusion [9].

Hydrogels, particularly those derived from natural polymers such as alginate, gelatin, and chitosan, are widely utilized in bioprinting because of their biocompatibility and ability in an aqueous environment, which is essential for cell viability and growth [10,11]. Hydrogels are hydrophilic, 3D polymeric networks characterized by high flexibility and capacity to absorb and hold substantial amounts of water while maintaining mechanical stability [1,12]. Physical hydrogels have several advantages, such as easy preparation and sol–gel transition [12,13]. However, such hydrogels often exhibit mechanical weaknesses in terms of gelation and printability, which limit their application in additive manufacturing processes. Therefore, significant efforts have been directed toward enhancing the mechanical properties of hydrogels, thereby improving their printability and functionality in tissue engineering. For instance, using a composite approach, alginate–gelatin composite bioinks, together with the incorporation of nanoparticles, have significantly improved mechanical strength, enhancing printability and cellular support in tissue engineering applications [14].

There are numerous challenges associated with working with conventional hydrogels, such as dynamic rheological behavior under different printing conditions, which leads to a loss of printing fidelity and structural integrity post-printing [15,16]. To address such challenges, efforts such as the use of composite polymers and blending of inorganic nanoparticles with biopolymers are being made for structural and functional enhancement. In addition, the use of sacrificial copolymers and printing into sacrificial support baths, whereby the sacrificial copolymer and support bath are removed after printing, is being actively investigated [17,18]. Furthermore, tissue engineering applications that cultivate electrically excitable cells, such as neurons, skeletal myocytes, and cardiomyocytes, could benefit from enhanced cell adhesion, cell–cell communication, proliferation, and differentiation when the hydrogel is conductive [19,20]. However, a critical research question remains to be answered: how can we engineer a hydrogel that retains the biocompatibility, printability, and rheological properties of alginate while enhancing its electrical conductivity properties?

Research on conductive hydrogels is advancing because of their potential applications in various fields including bioelectronics, drug delivery systems, tissue engineering, and biosensors [3]. Despite these advances, current hydrogel-based bioinks fail to fully support the cultivation and electrical stimulation of electrically excitable cells, such as neurons and cardiomyocytes, owing to their insufficient electrical conductivity. This limits the potential of 3D bioprinted tissues to mimic the native electroactive tissues. Therefore, continuous research efforts have been directed toward incorporating conductive materials into hydrogel bioinks by doping hydrogels with intrinsically conductive materials (ICMs) such as polypyrrole, poly(3,4-ethylenedioxythiophene): poly(styrene sulfonate) (PEDOT: PSS) [21,22], graphene oxide [23], polythiophene, and nanoparticles. While the incorporation of ICMs into hydrogels has been explored and shown to enhance the electrical properties while maintaining the unique characteristics of the hydrogels, such as flexibility and biocompatibility [23], conductive hydrogels prepared by doping ICMs present a significant setback: uncontrolled leaching of the conductive unit from the hydrogel owing to the non-covalent nature of the incorporation [24]. Thus, we hypothesized that covalently binding the intrinsically conductive unit to the alginate polymer could lead to a stable 3D printable electrically conductive hydrogel.

Sodium alginate is an ideal biopolymer candidate because of its excellent biocompatibility, non-toxicity, and ability to rapidly form 3D hydrogels upon interaction with divalent cations [25]. Furthermore, its thixotropic properties, including its shear-thinning behavior and viscosity recovery [26], make it particularly suitable for 3D printing applications, either as a standalone material or in combination with other polymers.

Despite their documented advantages, alginate hydrogels, like other biopolymer-based hydrogels, lack sufficient electrical conductivity, which limits their widespread application in bioelectronics, biosensors, and electroactive tissue scaffolds. Thus, we propose the integration of polypyrrole into alginate hydrogels to confer electrical properties to alginate while maintaining its rheological features, making it amenable to 3D bioprinting. This study introduces a new approach for creating an electrically conductive alginate-based composite hydrogel by covalently binding sodium alginate to a pyrrole monomer, from which conductive polypyrrole is chemically synthesized, either in situ or ex situ. Specifically, an *N*-substituted pyrrole derivative, aminopropyl pyrrole, was synthesized and conjugated with alginate using carbodiimide chemistry. The resulting alginate–pyrrole conjugate was characterized by its rheological properties, electrical conductivity, and 3D printability.

Wright et al. showed the printability of alginate mixed with a pyrrole monomer, which was subsequently polymerized into polypyrrole, and reported uncontrolled leaching of polypyrrole and brittleness of the resulting hydrogel [24]. Therefore, two strategies were explored in this study to incorporate polypyrrole into the alginate matrix: (1) oxidative polymerization of pyrrole directly within the 3D printed alginate–pyrrole network (in situ polymerization), referred to as alginate–PPy, and (2) pre-synthesis of polypyrrole nanoparticles (PPy-NP), which were subsequently blended with alginate (alginate@PPy-NP) prior to 3D printing. To the best of our knowledge, this is the first time that this approach has been employed to create an alginate–polypyrrole-based conductive hydrogel. The effects of these strategies on the printability, mechanical integrity, and electrical properties of hydrogels were examined. Notably, the electrical conductivity increased with the pyrrole content in both cases, and both types of alginate–polypyrrole were 3D printable, while alginate mixed with already-made polypyrrole nanoparticles displayed appreciably higher compressibility, without leaching out of the ICMs, highlighting the potential of this covalently functionalized alginate–pyrrole as an electrically conductive bioink.

## 2. Materials and Methods

### 2.1. Materials

All chemicals and reagents used in this study were of analytical grade and used without further purification, unless otherwise specified.

For alginate modification and hydrogel fabrication, sodium alginate (low viscosity, A2158), 1-ethyl-[3-(dimethyl amino)propyl]-3-ethyl carbodiimide HCl (EDC, E-1769), N-hydroxysulfosuccinimide (sulfo-NHS, 24510), 1-(2-cyanoethyl)-pyrrole, 2-[N-morpholino] ethane sulfonic acid (MES) buffer (M-8250), ammonium persulfate, phosphate-buffered saline (PBS) (P4417), sodium chloride, and calcium chloride (C-5426) were purchased from Sigma-Aldrich (Saint Louis, MO, USA). 3-(Aminopropyl) pyrrole was synthesized in-house as described in Section 2.2.

For nanoparticle synthesis, the pyrrole monomer (8.07492, 98% *w*/*v*) was distilled using a Heidolph rotavapor (Schwabach, Germany) and stored under nitrogen/argon at −80 °C until use. Ammonium persulfate (APS, ≥98%) was used as the oxidant for polypyrrole synthesis. Lithium aluminum hydride (LiAlH_4_, 212792) was employed as the reduction step in the synthesis of 3-(aminopropyl)pyrrole. All the reagents were obtained from Sigma-Aldrich (Saint Louis, MO, USA).

For crosslinking and 3D printing, calcium chloride (150 mM) was used as the ionic crosslinker. Gelatin (Type A, bloom 300) was obtained from Sigma-Aldrich (USA) and used in the preparation of the support bath for the FRESH (Freeform Reversible Embedding of Suspended Hydrogels) technique. Dulbecco’s Modified Eagle’s medium (DMEM) was purchased from Biological Industries (Kibbutz Beit-Haemek, Israel).

For instrumentation and imaging: Deuterated chloroform (CDCl_3_,151823) and methanol-d_4_ for NMR spectroscopy were obtained from Sigma-Aldrich (USA). Acetone, methanol, isopropyl alcohol (IPA), and deionized water were used to clean the electrode surfaces and were purchased from Bio-Lab (Ashkelon, Israel).

### 2.2. Synthesis of Aminopropyl Pyrrole

A solution of 1-(2-cyanoethyl) pyrrole (0.02 mol) was added dropwise to anhydrous ether (15 mL) in a suspension of LiAlH_4_ (0.05 mol) prepared in anhydrous ether (150 mL), refluxed for 10 h, and cooled. The excess hydride was quenched by successive addition of water (1.7 mL), a solution of 15% (*w*/*v*) NaOH (1.7 mL), and water (5.1 mL), and then heated at 40 °C for two h. The resulting solution was filtered through celite and the filtrate was evaporated to dryness. The synthesis of N-(3-aminopropyl)-pyrrole was confirmed by ^1^H NMR (400 MHz, CDCl_3_) δ: 6.70 (s, 2H, H–α), 6.24 (s, 2H, H–β), 3.25 (t, J = 6.8 Hz, 2H, –CH_2_–NH_2_), 2.65 (t, J = 7.2 Hz, 2H, –CH_2_–CH_2_–NH_2_), 1.85 (m, 2H, –CH_2_–CH_2_–CH_2_–) (Supplementary Materials Figure S1), FTIR, and mass (Supplementary Materials, Figure S2) spectroscopies.

### 2.3. Synthesis of Alginate–Pyrrole

**Figure 1:**
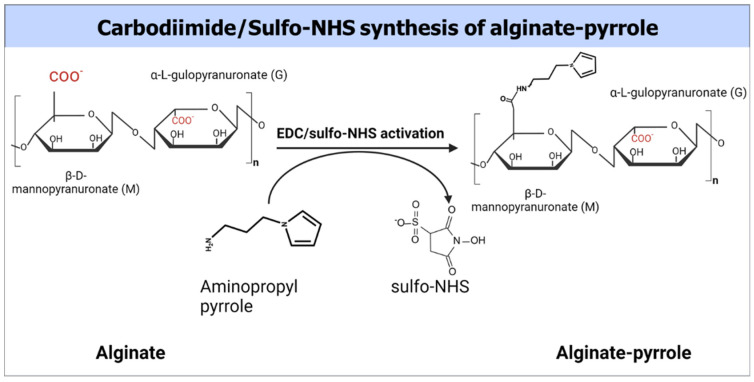
Covalent conjugation of pyrrole monomers to sodium alginate.

Na-alginate solution (2.5% *w*/*v*) was prepared by dissolving 500 mg of alginate in 20 mL of double-distilled water (DDW) with a resistivity of 18.2 MΩ and sterile filtered through a 0.22 µm filter (Stericup and Steritop EMD Millipore Corp., Billerica, MA, USA). To the filtered alginate solution, 852 mg of MES buffer and 400 mg of NaCl were added, and the mixture was stirred thoroughly. The pH of the solution was adjusted to 6.5. The carboxyl groups of sodium alginate were then activated using 1 mmol EDC and 0.5 mmol NHS to achieve a theoretical 50% molar modification relative to the total number of alginate carboxyl groups. The resulting solution was stirred for 3 h at room temperature. Subsequently, 480 mg (3 mmol) N-(3-aminopropyl) pyrrole was added to the activated alginate solution and stirred overnight at room temperature (Figure 1). The resulting polymer composite was dialyzed against doubly distilled water using a 3.5 kDa MWCO membrane (Spectrum Lab Membrane Filtration Products, Inc., Irving, TX, USA), with the water changed twice a day for three days. The dialysate was then lyophilized. The extent of pyrrole incorporation was determined by UV-vis spectroscopy using a pedestal mode of NanoDrop ONE^C^ desktop spectrophotometer (Thermo Fisher Scientific, Wilmington, DE, USA). Briefly, the extent of alginate modification by *N*-(3-aminopropyl)-pyrrole was determined by dissolving the alginate–pyrrole samples to produce a 0.01% (*w*/*v*) alginate–pyrrole solution and measuring the absorbance. The extent of alginate modification was determined from the calibration curve by measuring the absorbance of different amounts of *N*-(3-aminopropyl)-pyrrole in a 0.01% (*w*/*v*) Na-alginate solution. A standard solution of Na-alginate at a concentration of 0.01% (*w*/*v*) was used as blank (Supplementary Figure S5).

### 2.4. Attenuated Total Reflection Fourier-Transform Infrared Spectroscopy (ATR-FTIR)

Infrared spectra of the synthesized aminopropyl pyrrole and alginate–pyrrole were recorded using a Smart iTR^TM^ Diamond ATR-FTIR sampling accessory and Nicolet 6700 spectrometer (Thermo Fisher Scientific, Waltham, MA, USA) to characterize the functional groups of the synthetic products. All measurements were performed using a built-in diamond-attenuated total-reflection (ATR) crystal. The FTIR spectra were acquired over the range 4000–650 cm^−1^ at a resolution of 4 cm^−1^ and represented an average of 36 scans. The spectrum of the clean, dry diamond ATR crystal in ambient atmosphere (air) was used as the background for infrared measurements. The presence of pyrrole, amine, and amide groups was assessed by identifying the characteristic C=C, C–N, and N–H vibrations for pyrrole, N-H and C-N bonds for amines, and C=O and N–H bonds for amides.

### 2.5. X-Ray Photoelectron Spectroscopy (XPS) Analysis

X-ray photoelectron spectroscopy (XPS) analysis was conducted on lyophilized samples of alginate–pyrrole and alginate using an “ESCALAB Xi+” instrument (Thermo Fisher Scientific, Waltham, MA, USA). The ionization energies of oxygen, nitrogen, and carbon present in the lyophilized samples were recorded as previously described for alginate [27]. The analysis was performed at an ambient pressure of <1 × 10^−10^ mbar. An aluminum Kα radiation source (1486.68 eV) was used for photoelectron emission at room temperature, and spectra were collected at an angle of 90° from the X-ray source. A low-energy electron flood gun was employed to minimize surface charging and measurements were performed with a spot diameter of 650 µm. Spectral analysis was performed using Avantage software version 6.6.0, provided by Thermo Fisher Scientific. High-resolution spectra of the C1s, O1s, and N1s peaks and a survey spectrum at a pass energy of 20 eV were obtained.

### 2.6. Thermogravimetric Analysis (TGA)

Thermogravimetric analysis (TGA) was conducted to assess the thermal stability of sodium alginate (Na-alginate) and the synthesized Na-alginate–pyrrole (alginate–PPy) composite. A known mass of lyophilized samples of sodium alginate (1.598 mg) and sodium alginate–pyrrole (2.745 mg) was accurately weighed in an open alumina crucible and analyzed using TA Instruments Q500 and Q50 thermogravimetric analyzers (New Castle, DE, USA). The samples were heated from 20 to 600 °C at a constant rate of 10 °C/min under a nitrogen atmosphere (flow rate: 60 mL/min). The resulting thermograms were used to compare the decomposition profiles and residual mass of the unmodified and functionalized alginate materials.

### 2.7. Chemical Synthesis of Polypyrrole Nanoparticles (PPy-NP)

Polypyrrole (PPy) was synthesized via chemical oxidative polymerization using ammonium persulfate (APS) as the oxidant with a monomer-to-oxidant molar ratio of 1:1.5. The final APS concentration was 212 mM and the reaction was conducted at 4 °C for 120 min. Briefly, 1.0 mL of pyrrole monomer (14.4 mmol) was added to 50 mL of ice-cold deionized water (DDW) under continuous stirring. Separately, 4.83 g APS (21.6 mmol) was dissolved in 10 mL DDW and added dropwise to the pyrrole solution. The total reaction volume was adjusted to 100 mL using DDW and the mixture was magnetically stirred (260 rpm). Aliquots of 5 mL were withdrawn at defined time intervals to monitor the polymerization progress via UV-Vis spectroscopy at 460 nm (Supplementary Figure S4). Following polymerization, a black precipitate of PPy was collected by vacuum filtration, thoroughly washed with DDW and ethanol to remove residual monomers and oxidants, and dried under vacuum at 50 °C overnight.

#### Characterization of PPy-NPs

The surface morphology of the synthesized PPy was examined using field-emission scanning electron microscopy (FESEM, Thermo Fisher Verios 460 L, Waltham, MA, USA) at different magnifications. The samples were sputter-coated with gold and imaged at various magnifications to assess the surface features and particle aggregation.

Dynamic light scattering (DLS) analysis was carried out using an ALV/CGS-3 Compact Goniometer (Hessen, Germany) by dispersing 1 mg of dried PPy-NP powder in 1 mL of 1-methyl pyrrolidone and sonicating for 10 min in a bath sonicator (Bransonic 1210 Branson Heating Water Bath Ultrasonic Cleaner 1210R-MTH, Frankfurt am Main, Germany) to ensure uniform dispersion. The suspension was filtered through a 0.45 µm syringe filter to remove aggregates prior to measurement.

### 2.8. Preparation of Alginate–Pyrrole as Bioink

#### 2.8.1. Alginate–Pyrrole/Pyrrole (Alginate–Py) as Bioink

To create an optimal ink formulation, different stoichiometric ratios of pyrrole in alginate were prepared (Table 1) using double-distilled water (DDW) with a resistivity of 18.2 MΩ. To enhance the electrical conductivity, a defined amount of pyrrole monomer (14.125 M) was added to the alginate–pyrrole ink formulation prior to polymerization. This supplementation ensured a sufficient pyrrole concentration for in situ polymerization, as sub-threshold levels typically yield nonconductive oligomers. The dual inclusion of a covalently grafted pyrrole and a free monomer was designed to promote homogeneous polypyrrole formation throughout the hydrogel matrix. Lyophilized alginate–pyrrole and sodium alginate were separately dissolved in double-distilled water to a final concentration of 2.5% (*w*/*v*) under stirring for 2 h. Primary (partial) crosslinking of the alginate was achieved by mixing 1.2 mL of alginate–pyrrole, 1.2 mL of alginate, 0.3 mL of CaCl_2_ (0.1 M), and 0.021 mL of pyrrole monomer (14.125 M), using a homogenizer to equally distribute the calcium ions throughout the solution (Table 1). The mixture was then stirred at RT until further use. The partially crosslinked alginate–pyrrole/pyrrole was transferred to a sterile 3D printer syringe.

For the cell-laden bioink, 0.28 mL of Dulbecco’s Modified Eagle Medium (DMEM) containing 2.6 × 10^6^ cells/mL of isolated GFP-expressing fibroblasts mixed with DMEM was loaded into an additional sterile syringe, which was immediately connected to the alginate–pyrrole/pyrrole-loaded syringe through a Luer-to-Luer connector. The solutions were mixed by gently pushing the pistons back and forth for 1 min until homogeneous mixing was achieved. The final bioink consisted of 2% alginate–pyrrole (*w*/*v*), 0.01 M CaCl_2_ (or 0.36% *w*/*v* calcium gluconate), and 2.6 × 10^6^ cells/mL.

#### 2.8.2. Alginate@polypyrrole Nanoparticles (Alginate@PPy-NP) as Bioink

The possibility of blending PPy-NPs into a Ca-alginate hydrogel as a bioink (alginate@PPy-NPs) for 3D printing was explored. Different solutions of 3 mL (2% *w*/*v*) Na-alginate containing PPy-NP at final concentrations of 1, 0.33, and 0.17% *w*/*v*, corresponding to solutions with PPy-NP/alginate ratios of 8.3, 16.7, and 50% *w*/*w*, respectively, were prepared. The primary crosslinking was performed by dropwise addition of 0.05 M CaCl_2_ to reach a final concentration of 0.01 M and was stirred overnight and subsequently used for 3D printing or further processed for rheological testing and electrical conductivity testing.

### 2.9. Three-Dimensional Printing of Alginate–Pyrrole Conjugate and Alginate@polypyrrole Composites

Basic 3D models of the scaffold constructs were designed and spliced using Perfectory RP and software supplied by the Envision TEC 3D bioplotter. The designs were printed using a 3D Bioplotter Manufacturer Series system (EnvisionTEC GmbH, Marl, Germany) using VisualMachine BP interface software (V2.2; EnvisionTEC GmbH). Scaffolds were 11 mm × 11 × 2.5 mm with an inner strand distance of 2.25 mm and inner strand angles of 0° and 90°. The strands were dispensed layer-by-layer using pneumatic pressure in a piston-based system through 25-gauge blunt dispense needles (EFD Nordson, Wallisellen, Switzerland). The printing parameters were optimized in terms of the printing speed and pressure in the range of 10–22 mm per second and 0.5–1.0 bar, respectively.

Prior to bioprinting, as previously described [18], the bioink was deposited into a sterilized 30 mL printer cylindrical cartridge sealed with a fitted plunger utilizing a Luer-to-Luer connector. The printer cartridge was subsequently sealed and centrifuged for 1 min at 150× *g* to eliminate any remaining air bubbles. A sterile 25-gauge needle tip was attached to the cartridge and a cap connecting the print head to the barrel was affixed. The barrel was placed in the low-temperature head of the bioprinter (EnvisionTEC 3D-bioplotter Developer Series, Marl, Germany), set to 25 °C. The bioink was allowed to equilibrate to the printing head temperature for 30 min before bioprinting. The 3D printing was conducted by extrusion over the range of 0.5–0.8 bar and printing speed of 10–22 mm/s, followed by secondary crosslinking with 0.15 M CaCl_2_. In addition, the possibility of printing using the freeform reversible embedding of suspended hydrogels (FRESH), as described by Hinton et al. [17], was evaluated. The recipe and preparation of the FRESH can be found in Supplementary Material Protocol S1.

To eliminate the gelatin support bath, bioprinted constructs were incubated for 1 h at 37 °C and 5% CO_2_. The constructs were then washed and incubated for 10 min at 37 °C in DMEM supplemented with 0.1 M CaCl_2_ to further crosslink the 3D-bioprinted constructs, after which the constructs were incubated in DMEM supplemented with 20% fetal bovine serum (FBS) in a new 12-well plate.

#### Semi-Quantification of Printability

The printing factor (Pf) of the calcium alginate–polypyrrole inks to achieve square-shaped pores was quantified from light microscopy images using ImageJ software (ImageJ 1.54f, Java 1.8.0._322 64-bit) by comparing the measured area of the printed pores to the theoretical design. This parameter, adapted from previous studies on grid-based constructs, provides a quantitative indication of pore shape fidelity and filament uniformity [28]. The printability of the bioink was determined according to the method described by Ouyang et al. [28], based on the understanding of the circularity (C) of an enclosed area. The circularity of an enclosed area is defined as

(1) $$C=\frac{4\pi A}{L^{2}},$$

where L is the perimeter and A is the area.

The circles exhibit the highest circularity (*C* = 1). The closer the value of C is to one, the closer the shape is to a circle. For a square shape, the circularity is *π*/4 [28]. Because the model designed in this study was square, the bioink printability (Pr) based on the square shape was defined according to the following equation [28]:

(2) $$\mathrm{Pr}=\frac{\pi }{4}\;\frac{1}{C}=\frac{L^{2}}{16A}\;$$

In this study, the printability factor (Pf) is defined as

(3) $$\mathrm{Pf}=\frac{area\;of\;the\;square\;box\;printed\;}{area\;of\;the\;square\;box\;designed\;}$$

In this context, three statuses of Pf are defined as follows: Pf < 1 corresponds to under-gelation and typically rounded pore corners because of the fusion of layers and swelling; Pf = 1 corresponds to proper gelation with ideal square-shaped pores; and Pf > 1 corresponds to over-gelation, causing shrinkage of the construct [29]. Therefore, a Pf value close to 1 indicates a high degree of shape retention.

### 2.10. Rheological Characterization

The viscoelastic behavior of calcium alginate and calcium alginate–pyrrole hydrogels was assessed using a stress-controlled advanced rheometer (AR 2000; TA Instruments). A 40 mm diameter 3°58 steel cone geometry with a truncation gap height of 104 μm was used. The storage modulus (G′), loss modulus (G″), and complex viscosity (|η∗|) were recorded as a function of time through oscillatory measurements in a frequency range of 0.1–10 Hz, and the strain was determined to be in the linear viscoelastic region. In addition, the apparent viscosities (Pa·s) of the partially crosslinked hydrogels were assessed at shear rates between 0.1 and 100 s^−1^. Compressibility was measured using a rheometer (TA Instruments, model AR 2000) operated in flat-plate mode with a 40 mm diameter in an increasing normal force. Time and frequency sweep tests were conducted at a constant strain of 1%, which was within the linear viscoelastic region, as determined by preliminary strain amplitude tests. The applied stress during the time sweep measurements ranged between 0.5 and 1 Pa depending on the formulation, ensuring linear viscoelastic behavior throughout. Rheological measurements were performed with a sample size of n = 1 due to the large volume requirements of the rheometer and limited availability of material for each formulation.

#### Rheological Data Analysis

The steady-state flow behavior of the hydrogel formulations was analyzed by fitting the shear stress (τ) versus shear rate (γ˙) data to the Power Law model (Ostwald–de Waele equation) as follows:

τ = K ⋅ γ˙^n^ (4)

where K is the consistency index (Pa·s^n^) and n is the flow behavior index (dimensionless). Log–log linear regression was performed using OriginPro 2024 (OriginLab Corp., Northampton, MA, USA) to obtain the slope (n) and intercept (log K) of each material. The flow behavior index indicates the degree of shear thinning, with n < 1 confirming pseudoplastic behavior. The yield stress was approximated as the shear stress corresponding to the lowest non-zero shear rate.

For instance, the alginate control exhibited a shear-thinning index of n = 0.283 and a consistency index of K = 18.01 Pa·s^n^, indicating strong shear-thinning behavior. The yield stress was estimated to be 13.24 Pa, corresponding to a shear rate of 0.1 s^−1^. Complete rheological data can be found in the Supplementary Materials.

### 2.11. Electrical Conductivity Characterization

Electrical measurements were performed using a sandwich configuration at room temperature. Partially crosslinked calcium alginate, alginate–Py (later polymerized to alginate–PPy after crosslinking), and alginate@PPy-NP hydrogels were individually cast into custom-made molds and fully crosslinked overnight by the addition of 150 mM calcium ions. Each hydrogel sample (volume = 1 cm^3^) was placed between two stainless-steel plates (1 × 1 cm^2^), with the bottom plate acting as the working electrode and the top plate serving as the contact electrode.

Prior to assembly, the stainless steel plates were sequentially cleaned by sonication in acetone, methanol, isopropyl alcohol (IPA), and deionized water (DDW) for 15 min each, followed by nitrogen drying. The assembled device was positioned in a probe station (JANIS ST-500, Woburn, MA, USA) and secured by applying a vertical pressure to the top probe.

Current–voltage (I–V) measurements were conducted using a Keithley 2635 Source-Meter Unit (Cleveland, OH, USA), with data acquisition controlled by a custom MATLAB program (version 23.2.0.2365128 (R2023b)). The measurements spanned a voltage range from −1 V to 1 V in 0.05 V increments. The resistance (R_b_) was calculated from the linear slope of the I–V curve. The electrical conductivity (σ) was computed using the following equation:

(5a) $$R_{b}=\frac{L}{\sigma *A}$$

(5b) $$\sigma \;(µS/cm)=\left(\frac{L}{R_{b}*A}\right)\times 10^{6}$$

where σ is the conductivity (µS/cm), L is the hydrogel thickness (m), A is the contact area (m^2^), and R_b_ is the bulk resistance (Ω). The hydrogel dimensions were measured using a digital caliper, and all measurements were performed at room temperature under ambient conditions. All measurements were performed at room temperature, and a minimum of three replicates were tested per sample type for statistical validity.

### 2.12. Statistical Analysis

A two-way or one-way ANOVA, followed by a Tukey multiple comparisons test, was conducted using GraphPad Prism version 8.0.2 for Windows (GraphPad Software, San Diego, CA, USA, www.graphpad.com, accessed on 26 November 2024).

## 3. Results

### 3.1. ATR-FTIR and NMR Spectra of Aminopropyl Pyrrole

**Figure 2:**
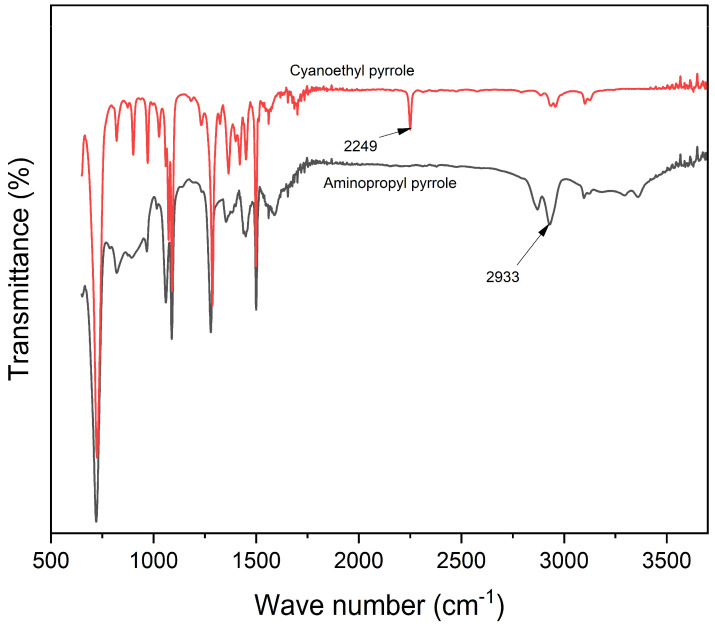
ATR-FTIR spectra of aminopropyl pyrrole.

Aminopropyl pyrrole is composed of a primary amine (NH_2_), repeated CH_2_ units, and a pyrrole ring. Primary amines undergo stretching (scissoring) and bending (wagging). As shown in Figure 2, the peaks at 3325, 2929, and 1278, respectively, are assigned to symmetric, asymmetric NH_2_ stretching, and C-N stretching, respectively. NH_2_ scissoring and wagging, which are detectable within 600–750 and 1630–1650 were found at 729 and 1627, respectively, while the in-plane and out-of-plane N-H bending appeared at 1567 and 720 cm^−1^, respectively [30].

The FT-IR spectra of sodium alginate and alginate–pyrrole were compared to confirm the structural transformation of Na-alginate by the incorporation of covalent pyrrole into alginate (Supplementary Figure S3). The broad band at approximately 3600–3060 cm^−1^ was assigned to hydrogen-bonded O-H stretching vibrations, and the two peaks at 2914 and 1591 were attributed to C-H stretching and C=O asymmetric stretching, respectively. The peak at 1404 cm^−1^ was assigned to both C-OH deformation and C=O asymmetric vibrations of the carboxylate groups [31]. In addition, additional peaks characteristic of alginate were observed at 1025 and 1053 cm^−1^, which were attributed to C-O stretching vibrations and C-O + C-C stretching vibrations of the pyranose rings, respectively. Additionally, the bands at 812 and 880 cm^−1^, respectively, were assigned to mannopyranuronic acid and α-L-gulopyranuronic acid asymmetric ring vibrations, respectively, and were attributed to the α1-4 bond [31,32]. The pyrrole monomer has certain bonds whose vibrational state changes during IR spectroscopy and occurs in identical regions in the electromagnetic spectrum as Na-alginate and pyrrole ring deformation at 730 cm^−1^.

For instance, the N-H in-plane stretching, C-C in-ring stretching modes, and ring vibrations of pyrrole have been reported to occur at 3396, 1571, and 1467 cm^−1^, respectively, which overlap with some regions previously assigned to sodium alginate [33,34]. In addition, the peaks attributable to amide bonds formed during alginate–pyrrole conjugation are reported to be located at approximately 1650 cm^−1^, which overlaps well with the C-H stretching of the carboxylate groups of alginates. Therefore, it is desirable to distinguish between pyrroles and alginate. Such a feature is the peak detected at 729 cm^−1^ in the alginate–pyrrole and aminopropyl pyrrole spectra but absent in the Na-alginate spectrum. This peak is attributed to the C-H vibration of the 2,5 hydrogen atoms of the pyrrole ring [33].

The ^1^H-NMR spectrum revealed different hydrogens in the synthesized aminopropyl pyrrole (Supplementary Figure S1). Some hydrogens attached to a carbon appeared at ~0.5–5.0 ppm, while other hydrogens attached to an amine appeared at ~2.3–3.0 ppm. The latter hydrogens were de-shielded due to the electron-withdrawing effects of nitrogen and appeared downfield in the NMR spectrum compared with the alkane hydrogens. This finding was further corroborated by the ^13^C NMR spectrum of the synthesized aminopropyl pyrrole (Supplementary Figure S1).

### 3.2. X-Ray Photoelectric Spectroscopy (XPS) of Alginate–Pyrrole

**Figure 3:**
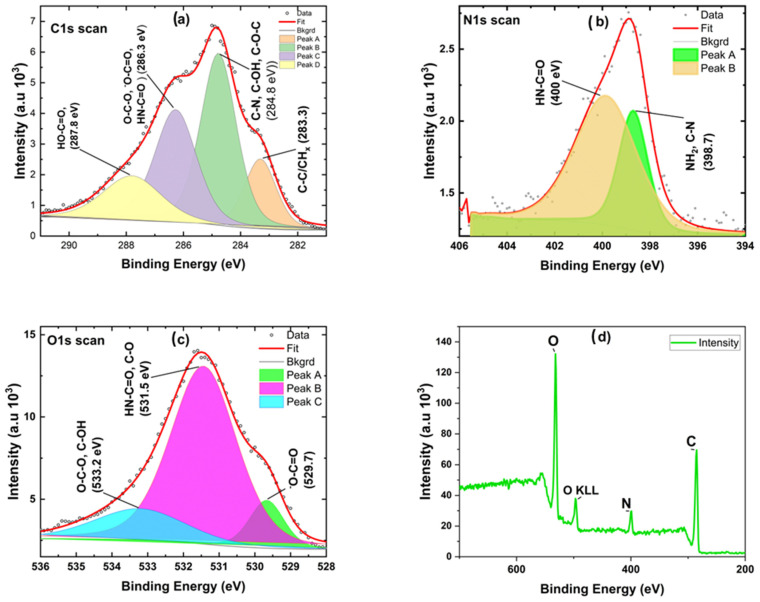
Comparison of deconvoluted XPS spectrum peaks (**a**–**d**) and survey analysis (**d**) of lyophilized alginate–pyrrole (alginate–Py). The deconvoluted peaks were assigned to chemical groups based on the binding energies of the peaks (N1s, O1s, and C1s).

XPS was used to quantitatively determine the elemental and chemical compositions of Na-alginate and synthesized alginate–pyrrole. High-resolution spectra were recorded for carbon, oxygen, and nitrogen, as shown in Figure 3. The compositions were calculated from the XPS spectra recorded in survey mode and expressed as relative atomic percentages.

X-ray photoelectron spectroscopy (XPS) confirmed the covalent conjugation of 3-aminopropyl pyrrole to sodium alginate via amide bond formation in the carbodiimide mediated synthesis. This was established by evaluating changes in carbon, nitrogen, and oxygen chemical environments (Table 2, Figure 3). The deconvoluted XPS spectra and survey analysis of the unmodified sodium alginate are presented in the Supplementary Materials (Figure S4).

In the C1s spectrum, unmodified sodium alginate showed a carboxyl peak at 288.08 eV (7.1%, Scan D), attributed to -COOH/-COO^−^ groups. In the alginate–pyrrole conjugate, this peak shifted to 288.4 eV with a markedly increased atomic contribution (33.63%), consistent with the formation of amide carbonyl (O=C-N). For instance, it has been established that the C1s peak for carbonyl groups within amides (C=O) appears around 287.0 eV to 288.2 eV [35]. The intensity increase indicates a possible conversion of carboxyl groups to amide linkages. A reduction in the C1s Scan C region (~286.3 eV) from 20.23% in alginate to 13.62% in the conjugate suggests redistribution due to C-N bond formation.

The N1s spectrum, absent in sodium alginate, appeared in the conjugate with peaks at 399.5 eV (4.43%) and 402.8 eV (3.67%). The 399.5 eV peak corresponds to pyrrolic nitrogen (C-N=C) [36] while the 402.8 eV peak is attributed to amide nitrogen (-CONH-) [37], possibly influenced by protonation or hydrogen bonding. In amide nitrogen, the N1s binding energies are often reported between 399.5 eV and 400.2 eV, often depending on the surrounding chemical environment [38]. In the work of Biemolt et al., the N1s peak was aligned to around 399.0 eV [38]. Furthermore, from the existing literature, it is well-established that pyrrole presents different nitrogen species contributing to the XPS N1s spectrum. Specifically, the binding energy associated with pyrrolic nitrogen (-NH) is typically observed around 400.4 eV [39]. These peaks confirm nitrogen incorporation through amide formation.

In the O1s region, alginate displayed peaks at 529.76 eV (5.12%), 531.48 eV (27.79%), and 533.18 eV (13.5%), representing carboxyl C=O and C-OH groups. In the conjugate, peaks at 531.15 eV (10.02%) and 533.15 eV (8.52%) indicate a shift toward amide carbonyl oxygen, with a corresponding reduction in carboxyl oxygen intensity. For oxygen in amides, the O1s peak is typically found at around 532 eV when derived from amide groups [40]. This signal often overlaps with signals from other oxygen-containing functional groups, necessitating careful deconvolution to discern the contributions specifically from amide functionalities [40]. Studies have utilized comprehensive fitting models to accurately depict the overlapping peaks and improve the resolution of oxygen assignments. A minor peak at 536.56 eV (4.9%) may reflect trace impurities or new oxygen environments. The nitrogen-to-carbon (N/C) ratio increased from approximately zero in native alginate to 0.14 (8.10% N/58.46% C) in the conjugate, supporting successful conjugation of nitrogen-containing 3-aminopropyl pyrrole. Collectively, the emergence of amide-specific peaks, altered elemental distributions, and increased N/C ratio provide an evidence of amide bond formation in the alginate–pyrrole conjugate.

### 3.3. Thermogravimetric Analysis

**Figure 4:**
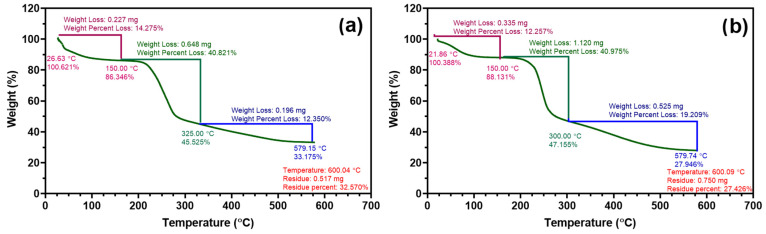
Thermogravimetric analysis (TGA) and derivative thermogravimetry (DTG) curves of (**a**) sodium alginate (Na-alginate) and (**b**) synthesized alginate–pyrrole (alginate–PPy) composites under nitrogen atmosphere. Both samples were heated from 20 to 600 °C at a rate of 10 °C/min. The TGA profiles revealed characteristic multistep degradation patterns, with alginate–PPy exhibiting an additional DTG peak associated with polypyrrole decomposition, and a slightly reduced residual mass compared to pristine alginate. These differences confirm the successful incorporation of PPy and its influence on the thermal stability. A summary of the weight decomposition is presented in the Supplementary Materials.

Thermogravimetric analysis (TGA) was performed to evaluate and compare the thermal stability of the pristine sodium alginate (Na-alginate) and the alginate–pyrrole (alginate–PPy) composite. The TGA and derivative thermogravimetric (DTG) profiles are presented in Figure 4.

Na-alginate displayed a three-step thermal degradation pattern. The initial weight loss (~14.28%) occurred between 30 and 150 °C, which was attributed to the evaporation of surface-adsorbed and bound water. A major decomposition step occurred between 150 and 325 °C, corresponding to the thermal degradation of the alginate backbone (i.e., depolymerization, decarboxylation, and cleavage of glycosidic linkages), with a mass loss of 40.82%. A subsequent minor decomposition stage occurred between 325 °C and 580 °C, accounting for an additional 12.35% mass loss. The final residual mass at 600 °C was 32.57%, which was likely associated with the formation of inorganic residues (e.g., sodium oxide) and carbonaceous char.

In contrast, the degradation profile of alginate–PPy was modified. The initial water loss (~12.26%) occurred at temperatures of up to 150 °C. A broad primary degradation event centered at 246 °C led to 40.98% weight loss, similar to that of pure alginate, but occurred at a slightly lower temperature, suggesting minor plasticization by PPy. Notably, a second DTG peak at ~397 °C (absent in pure alginate) confirmed the presence of polypyrrole domains, which thermally decomposed at elevated temperatures. The final residue at 600 °C was 27.43%, which was lower than that of the pure alginate. More details can be found in the Supplementary Materials (Figures S12 and S13).

### 3.4. Particle Size and Morphology of PPy-NPs

**Figure 5:**
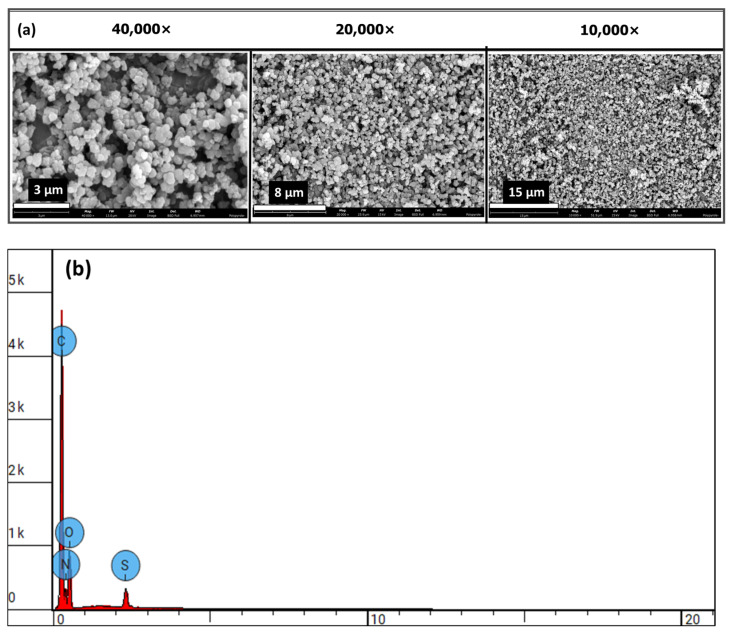
SEM micrographs of chemically prepared polypyrrole nanoparticles (PPy-NP). (**a**) The micrographs at different magnifications, (**b**) the EDS elemental analyses of the synthesized PPy-NP.

The polypyrrole nanoparticles (PPy-NPs) synthesized in this study via chemical oxidative polymerization using ammonium persulfate (APS) at 4 °C for 120 min were preliminarily characterized by SEM, which confirmed the formation of nanoscale particles with moderate aggregation, likely owing to the intrinsic hydrophobicity of PPy. Scanning electron microscopy (SEM) revealed the successful synthesis of polypyrrole nanoparticles (PPy-NPs) with uniform spherical morphology and submicron size distribution (0.29182 ± 0.06076 µm) (Figure 5). At increasing SEM magnifications (10,000× to 40,000×), the polypyrrole nanoparticles (PPy-NPs) displayed dense packing with moderately rough surfaces, suggesting their potential as reinforcing agents in hydrogel matrices. To better evaluate their size distribution, dynamic light scattering (DLS) analysis was performed under identical synthesis conditions. As shown in Supplementary Figure S11, DLS revealed a narrow, intense peak centered at a hydrodynamic radius of approximately 786 nm, which is notably larger than the ~300 nm average diameter observed by SEM.

This discrepancy is attributed to nanoparticle aggregation in suspension and the influence of the hydration shell captured by DLS. Transmission electron microscopy (TEM) further confirmed the presence of aggregated nanoscale particles with an average diameter ~296 ± 50 nm (Supplementary Figure S12), consistent with SEM observations. The incomplete dispersion of PPy-NPs in 1-methylpyrrolidone, even after ultrasonication, may have contributed to the elevated hydrodynamic size measured by DLS.

Collectively, the SEM, TEM, and DLS results affirm the nanoscale dimensions of the synthesized PPy-NPs while indicating partial aggregation in solution, a factor relevant to their incorporation in hydrogel formulations.

### 3.5. Rheological Behavior of Calcium Alginate–Pyrrole Hydrogels

A key requirement for the successful three-dimensional (3D) bioprinting of hydrogels is the capability of the bioink to undergo a liquid-to-gel phase transition. Bioink should be injectable and gel rapidly upon extrusion from its surface. Alginate crosslinks into the hydrogel through calcium ion-mediated electrostatic interactions, which determine the sol–gel phase-transition conditions. Additionally, components that modify crosslinking, such as partial covalent modification of the carboxyl group, affect the mechanical properties of the hydrogel, necessitating the characterization of the rheological properties of chemically functionalized physical calcium alginate hydrogels. The rheological data presented reflect single measurements (n = 1) per formulation, as restricted by instrument sample volume requirements and material availability.

#### 3.5.1. Viscosity and Stored (G′) and Loss (G″) Moduli

**Figure 6:**
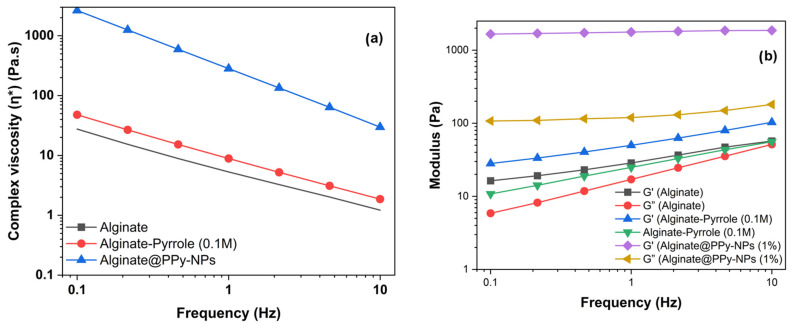
Oscillatory rheological behavior of calcium alginate, calcium alginate–pyrrole (0.1 M), and calcium alginate@PPy-NP (1%). (**a**) Complex viscosity (|η*|) as a function of frequency showing higher resistance to deformation in the modified formulations. (**b**) Storage modulus (G′) and loss modulus (G″) versus frequency. Calcium alginate@PPy-NP displayed the highest G′ across frequencies, indicating a superior elastic response and structural integrity.

To evaluate the rheological behavior of Ca-alginate and pyrrole-modified alginate (alginate–PPy and alginate@PPy-NP) hydrogels, primary crosslinking was performed at a final Ca^2+^ concentration of 10 mM (because primary crosslinking was performed prior to 3D printing). The mechanical properties of hydrogels, particularly their stored modulus (G’), indicate their ability to withstand deformation without permanent damage. For instance, hydrogels with G’ are significantly more significant than their loss modulus (G”), demonstrating a stable crosslinked structure, which is essential for maintaining shape and function under stress [41]. The time-dependent viscoelastic behavior of the alginate–PPy and alginate@PPy-NP formulations was evaluated using oscillatory rheology by monitoring the progression of the storage modulus (G′), loss modulus (G″), and complex viscosity (|η*|) as a function of time. As shown in Figure 6, both G′ and G″ gradually increased over time, and a sharp increase was observed after 60 s. Notably, G’ increased with the amount of pyrrole (Table 3 and Table 4) and the addition of ex situ generated polypyrrole nanoparticles to calcium alginate (calcium alginate@PPy-NP).

For the alginate–PPy system, the incorporation of pyrrole enhanced the gelation behavior compared to that of plain alginate. The alginate–PPy composite exhibited a marked increase in G′ from 28.11 Pa to 103.00 Pa, while viscosity complex (η*) decreased steadily from 47.24 Pa·s to 1.86 Pa·s over time, indicating the formation of a stable, elastic network. Alginate/pyrrole samples (0.05 M and 0.1 M) followed a similar but less pronounced trend, with a final G′ values of 45.25 Pa and 83.38 Pa, respectively. In contrast, plain alginate showed limited structural evolution, with G′ reaching only 57.05 Pa and G″ approaching parity. Conversely, the calcium alginate@PPy-NP system, which incorporated pyrrole nanoparticles at concentrations of 0.17, 0.3, and 1% (*w*/*v*), exhibited concentration-dependent enhancements in viscoelastic properties. The formulation containing 1% PPy-NP demonstrated the most significant elastic response, with the storage modulus (G′) increasing from 1660 to 1861 Pa, whereas the complex viscosity (|η*|) markedly decreased from 2648 to 29.76 Pa·s. Lower concentrations, specifically 0.3% and 0.17%, displayed similar, but progressively diminished trends. In contrast, the alginate control exhibited a minimal elastic structure.

Taken together, these results reveal that pyrrole-based modifications of alginate—both molecular (alginate–PPy) and particulate (alginate@PPy-NP) not only preserve but also enhance the time-dependent gelation and elastic behavior of conductive pyrrole-modified alginate, with potential implications for improved structural fidelity in 3D bioprinting applications.

The storage modulus (G′) and loss modulus (G″) of calcium alginate and its pyrrole composite hydrogels as a function of frequency (Hz) at 25 °C are shown in Supplementary Figure S7a–d for the calcium alginate–pyrrole (alginate–PPy) conjugate and Figure S7a–d for alginate mixed with polypyrrole nanoparticles (calcium alginate@PPy-NP). The G′ and G″ values of all the composite hydrogels increased with increasing frequency over the entire frequency range tested. Notably, the storage modulus G′ was greater than the corresponding loss modulus G″ over the entire frequency range, indicating the possible formation of a crosslinked structure by the hydrogels with good stability.

**Figure 7:**
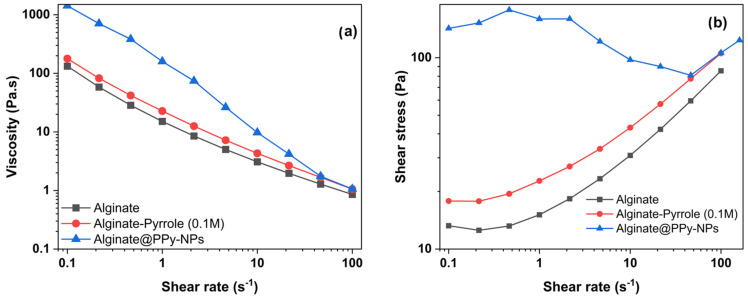
Steady-state rheological properties of alginate, alginate–pyrrole (0.1 M), and alginate@PPy-NP (1%). (**a**) Viscosity as a function of shear rate, showing pronounced shear-thinning behavior across all samples. (**b**) Shear stress versus shear rate profiles used to evaluate the yield stress and flow behavior of inks. Calcium alginate@PPy-NP exhibited the highest shear stress, which was consistent with the increased network resistance under flow.

The plot of viscosity against shear rate shows that all samples exhibit shear-thinning (non-Newtonian) behavior as the viscosity decreases with increasing shear rate (Figure 7). This is typical for polymeric and gel-like systems. Shear-thinning behavior was observed for all samples, and higher pyrrole concentrations led to increased viscosity and shear stress.

To enhance clarity and enable direct comparison across formulations, rheological parameters from the frequency sweep and steady-state flow analyses were compiled in summary tables. Table 3 presents the viscoelastic metrics (G′, G″, |η*|, and tan δ at 1 Hz), whereas Table 4 outlines the steady shear behavior, including viscosity at defined shear rates, yield stress, and shear-thinning index (n). These data capture the concentration-dependent modulation of the hydrogel mechanics and printability. The third table (Table 5) offers a condensed interpretation, correlating rheological performance with observed printing fidelity to support formulation selection for extrusion-based bioprinting.

To facilitate a comparative interpretation of rheological properties in relation to 3D printability, a condensed summary of each formulation’s rheological characteristics and the corresponding printability performance is provided in Table 5.

#### 3.5.2. Compressibility Test

**Figure 8:**
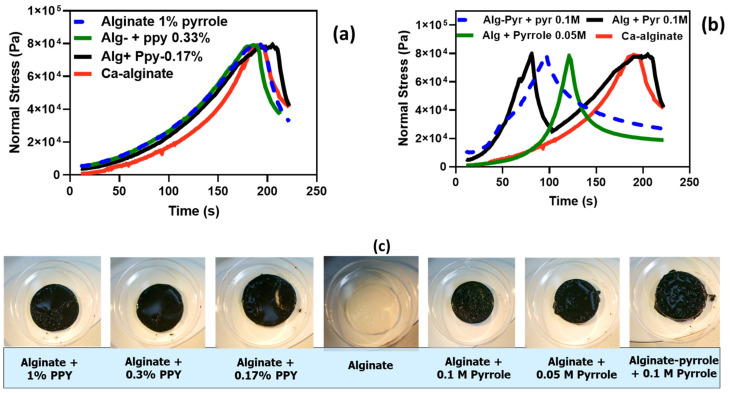
Compressibility test for (**a**) ex situ-generated alginate/polypyrrole composite, (**b**) in situ-generated alginate–polypyrrole composite, and (**c**) images of the composite hydrogels after the compressive stress was removed.

The mechanical properties of the composite hydrogels are shown in Figure 8a–c. The compressibility test was performed at increasing normal force (Pa) over time (s). Alginate and alginate with ex situ generated polypyrrole displayed a characteristic compressive stress of 80 kPa and a gradual permanent deformation when the time exceeded 180 s (Figure 8a). In addition, calcium alginate with an in situ generated polypyrrole had a characteristic compressive stress of 80 kPa and a sharp permanent deformation when the time exceeded 90 s, which is half the time required for a permanent deformation to set in the counterpart composite (Figure 8b). The lower the pyrrole content, the longer it takes to achieve a permanent deformation.

### 3.6. Electrical Conductivity

**Figure 9:**
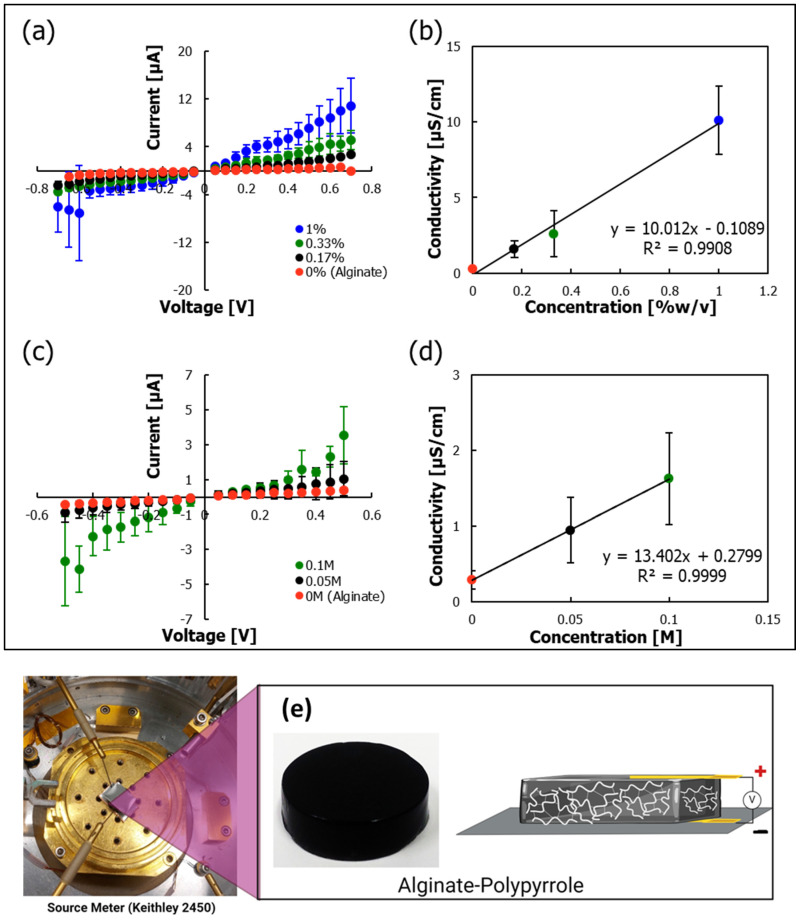
Electrical conductivity test. (**a**) Current–voltage plot of alginate@PPy-NP expressed as percentage (PPY %). (**b**) Plot of concentration-dependent conductivity of alginate@PPy-NP in percentage (PPY %). (**c**) Current–voltage plot of calcium alginate–PPy mixed with pyrrole monomer (expressed in molar, M) polypyrrole generated in situ. (**d**) Plot of concentration-dependent conductivity of calcium alginate mixed with pyrrole monomer (expressed in molar, M) polypyrrole generated in situ. (**e**) The schematic depiction of the assembly of hydrogel sample sandwiched between two stainless-steel plates with the bottom plate acting as the working electrode and the top plate serving as the contact electrode. The error bars in (**a**–**d**) represent the standard deviation of the statistical means from multiple measurements (n = 5 or more).

To investigate the electrical conductivity of the alginate–pyrrole samples, current–voltage curves were extracted for devices in which the hydrogel was sandwiched between stainless steel disks as contacts (Figure 9e). A linear increase in conductivity with an increase in pyrrole loading led to a proportional increase in conductivity (Figure 9b), which was extracted as the inverse of the slope of the current–voltage curve and was observed for both ex situ, calcium alginate@PPy-NP (Figure 9a,b), and in situ calcium alginate–PPy (Figure 9c,d) samples. This increase in conductivity can be attributed to electron transport within the pyrrole network. A conductivity of 10 µS/cm was achieved at a polypyrrole loading of 1% *w*/*v*. Polymerizing pyrrole within the calcium alginate–pyrrole conjugate led to a larger increase in conductivity compared to similar pyrrole loading levels (Supplementary Figure S10). However, the conductivity reached a maximum of 2 µS/cm at a pyrrole-loading level of 0.1 M. Notably, adding pyrrole to the pyrrole conjugate can also lead to a decrease in conductivity at higher concentrations [24]. The reduction in conductivity when a higher loading of pyrrole was used suggests a decrease in ion conductivity within the hydrogel [24]. Notably, the polymerization of pyrrole within the gel led to a higher level of water hydrolysis when higher voltages were applied to the sample.

### 3.7. Three-Dimensional Printability Characterization

**Figure 10:**
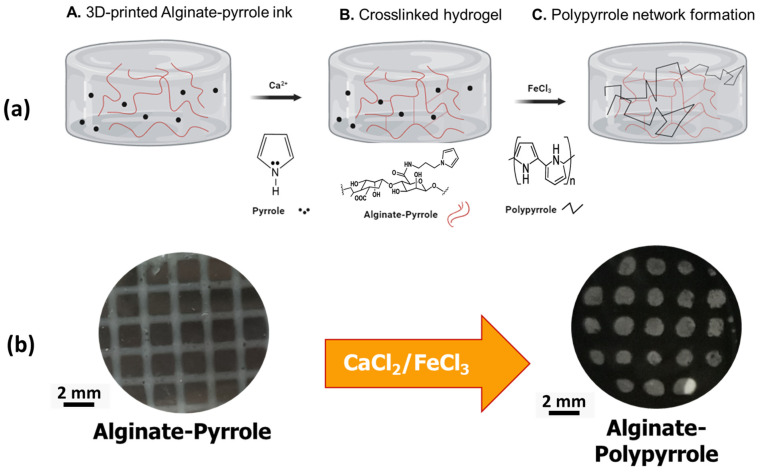
Scheme of post-printing steps (**a**) and image of printed grids followed by secondary crosslinking and in situ polypyrrole synthesis (**b**).

Bioink printability was assessed by evaluating the integrity of printed multilayer constructs. Three gelation states have been described for printed bioinks: under-gelation, proper-gelation, and over-gelation [28,29]. When the bioink is under ideal gelation conditions, the extruded structure should maintain constant and uniform filaments, resulting in a standard grid construct with distinguishable layers and dimensions that are similar to the designed layout. As shown in Figure 10, extrusion was followed by crosslinking with calcium ions and polymerization of the pyrrole anchored within the hydrogel.

**Figure 11:**
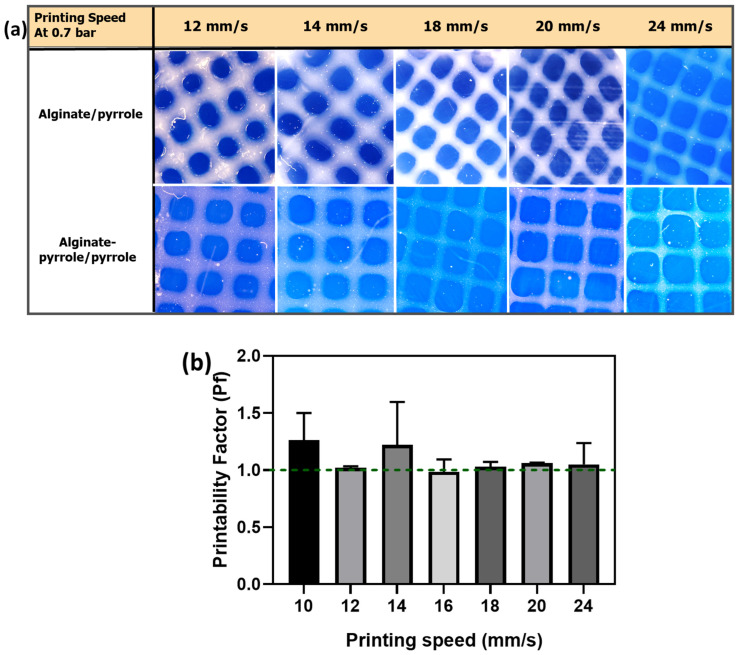
Printability studies as a function of printing speed at a printing pressure of 0.7 bar. (**a**) Images of the construct of calcium alginate–Py and calcium alginate–Py/pyrrole, and (**b**) the printability of calcium alginate–pyrrole/polypyrrole. No statistically significant differences (*p* < 0.05) were observed among the printability factors of the tested formulations. The dashed line shows the optimum printability factor.

The printing pressure and speed were evaluated during printing. Printability was calculated using Equation (2); the printability factor (Pf) was within 0.9–1.1 and considered acceptable, demonstrating structural and morphological stability. Figure 11a shows images of the various constructs of calcium alginate/pyrrole and calcium alginate–pyrrole/pyrrole extruded at a constant printing pressure (0.7 bar) and printing speed ranging from 12 mm/s to 24 mm/s. A plot of the printability factor with respect to printing speed is shown in Figure 11b. Printing speeds of 16, 18, and 20 mm/s consistently demonstrated the printability of different calcium alginate–pyrrole bioink formulations.

**Figure 12:**
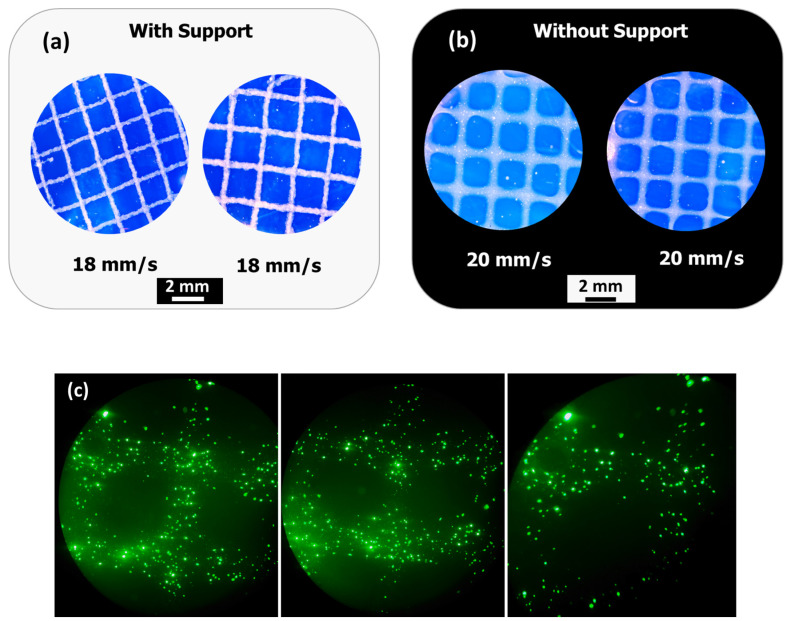
(Bio)Printing in a support bath (FRESH). (**a**) Construct printed with support, (**b**) construct printed without support, and (**c**) construct of bioink loaded with GFP-expressing human fibroblasts after removing FRESH. Note: A scale bar could not be provided for (**c**) because the image was captured without calibration.

The possibility of extruding the bioink directly into the support bath was evaluated using calcium-ion-supplemented free-form reversible embedding of suspended hydrogels (FRESH) as described by Hinton et al. [17]. As shown in Figure 12a, the construct was extruded into a support bath at a printing speed of 18 mm/s. After the sacrificial FRESH support was removed, images were captured after incubation at 37 °C for 60 min.

As shown in Figure 12b, the construct was extruded at a printing speed of 20 mm/s without a support bath followed by Ca^2+^ ion-mediated crosslinking. Figure 12a,b show the practicality of printing calcium alginate–pyrrole with or without support, depending on the application needs. Finally, calcium alginate pyrrole was loaded into GFP-expressing human fibroblasts and printed onto a calcium-containing FRESH support. After printing, the support was removed, and the cell-laden construct was incubated in fetal bovine serum (FBS)-supplemented DMEM in an incubator maintained at 37 °C and 5% CO_2_ (Figure 12c).

#### Printability of Alginate@PPy-NP Constructs

**Figure 13:**
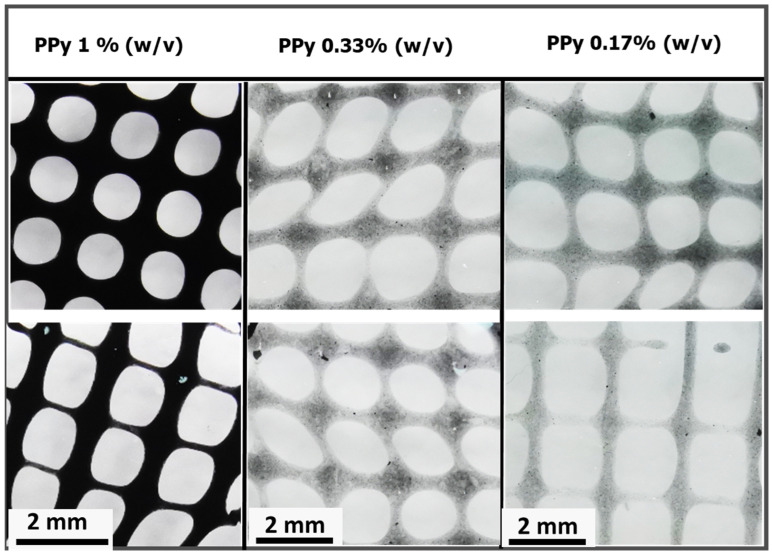
Light microscopy images showing grid constructs printed with Alginate@PPy-NP bioinks at different polypyrrole concentrations: 1% (*w*/*v*), 0.33% (*w*/*v*), and 0.17% (*w*/*v*). Extrusion-based 3D construct of calcium alginate@PPy-NP was carried out at a printing speed of 18 mm/s and pressure of 0.7 bar. The top and bottom rows show two representative grid constructs printed using the same bioink concentration to illustrate repeatability across samples. Increased PPy-NP (1% *w*/*v*) content is associated with sharper pore definition and reduced filament spreading.

Extrusion-based 3D printing of alginate@PPy-NP was performed at various printing pressures and speeds. The resulting constructs showed good printability across all the PPy-NP concentrations tested (Figure 13). At 1% (*w*/*v*) PPy-NP, the printed lines displayed appreciable structural fidelity, whereas lower concentrations (0.33% and 0.17%) resulted in reduced printing resolution and minor spreading at intersections, suggesting concentration-dependent improvements in shape fidelity and filament stability. These observations were in agreement with the enhanced rheological and mechanical properties observed at higher PPy-NP loadings.

## 4. Discussion

In the category of conducting polymers, polypyrrole (PPy) is widely used because of its low-cost preparation, excellent conductivity, and large surface area. PPy, a conductive polymer, facilitates the immobilization of various metallic nanoparticles on the surface of electrodes through π-π stacking, electrostatic interactions, or entrapment procedures [42]. In this study, we synthesized an N-substituted pyrrole (aminopropyl pyrrole) and its covalent conjugation to calcium alginate, which could be polymerized in situ into a calcium alginate–polypyrrole (calcium alginate–PPy) conjugate. In addition, polypyrrole nanoparticles (PPy-NPs) were chemically synthesized and blended with sodium alginate to produce a stable conductive alginate–polypyrrole composite (alginate@PPy-NP), which was used independently together with calcium alginate–PPy as inks for extrusion-based 3D printing.

Spectroscopic analysis confirmed the successful incorporation of PPy into the alginate matrix via both in situ polymerization and nanoparticle embedding. FTIR, mass spectrometry, and NMR (Supplementary Figures S1 and S2), confirmed the success of aminopropyl pyrrole synthesis, while X-ray photoelectron spectroscopy (XPS) revealed characteristic binding energies for C-C, C-O, and N-H and NH-C=O species, characteristic of amide bond formation (Figure 3), and ATR-FTIR (Supplementary Figure S3) confirmed the presence of polypyrrole and its interaction with the alginate backbone. From the existing literature, it is well-established that pyrrole presents different nitrogen species contributing to the XPS N 1s spectrum. Specifically, the binding energy associated with pyrrolic nitrogen (−NH) is typically observed around 400.4 eV [39]. Polypyrrole nanoparticles (PPy-NPs) were synthesized via chemical oxidative polymerization using ammonium persulfate (APS) at 4 °C for 120 min. The particle size of PPy-NPs is strongly influenced by several synthesis parameters, including monomer concentration, oxidant-to-monomer molar ratio, temperature, and stirring speed. Under the specified reaction conditions (monomer-to-oxidant ratio of 1:1.5, 4 °C, magnetic stirring), the particle size was typically in the range of 40–200 nm, as reported in the literature [43]. Mahmood et al. demonstrated that PPy-NPs synthesized via low-temperature oxidative polymerization could achieve sizes ranging from 85 to 300 nm, with the particle size being tunable by varying the reactant concentration [43]. Additionally, Effati et al. [44] reported the successful synthesis of PPy-NPs using a microfluidic system, highlighting that factors such as the oxidant-to-monomer molar ratio and reaction temperature significantly influence particle size and morphology [44]. Furthermore, a comprehensive review by Hao et al. discussed the control of PPy nanoparticle size and morphology, emphasizing the roles of synthesis conditions and the use of stabilizing agents [45]. These studies corroborate our reported size range of 40–300 nm for PPy-NPs synthesized under the specified conditions (monomer-to-oxidant ratio of 1:1.5, 4 °C, 260 rpm) and underscore the critical influence of the synthesis parameters on nanoparticle size control.

Rheological measurements demonstrated that both the alginate–PPy and alginate@PPy-NP systems exhibited time-dependent increases in the storage modulus (G′) and reductions in the complex viscosity (|η*|), indicating progressive gelation. The in situ polymerized calcium alginate–PPy system showed moderate improvements in viscoelasticity, whereas the alginate@PPy-NP system exhibited substantial enhancements, particularly at 1% (*w*/*v*) PPy-NP. A higher storage modulus (G′) indicates that the hydrogel possesses greater elasticity and structural integrity, which enables it to resist deformation and recover its original shape under mechanical stress. This characteristic is particularly important for applications that require mechanical stability and resilience, such as load-bearing or dynamic environments [46]. G′ is a key parameter for evaluating the elastic behavior of hydrogels and their suitability for structural and functional applications. For instance, Abd El-Aziz et al. demonstrated that incorporating borosilicate glass into gelatin-based hydrogels significantly enhanced the compressive modulus, resulting in an elastic material with G’ that is markedly higher than the loss modulus (G″), thereby confirming its mechanical robustness and ability to maintain a self-standing structure [47]. This characteristic is particularly beneficial in applications such as 3D printing and tissue scaffolding, where mechanical stability and resilience are necessary to support printing fidelity, cellular activity, and tissue regeneration [48]. The ability of hydrogels to maintain their structural integrity under load is further emphasized by studies showing that higher G’ correlates with increased gel strength and reduced water absorbency under pressure [48]. This concentration-dependent behavior suggests that the nanoparticles act as physical crosslinkers, promoting network densification and elastic recovery. Shear-thinning behavior was observed across all formulations, which is favorable for extrusion-based bioprinting.

The mechanical analysis using compressibility tests further supported these rheological findings (Figure 8). The Ca-alginate@PPy-NP hydrogels (Figure 8a) exhibited a clear concentration-dependent increase in the compressive strength and deformation resistance, with the 1% NP variant achieving the highest mechanical robustness. In contrast, alginate–PPy hydrogels displayed symmetric stress–strain profiles, indicative of homogenous, yet brittle networks. Notably, the deformation pattern of alginate@PPy-NP extended beyond 180 s, whereas alginate–PPy (Figure 8b) exhibited earlier yielding, reflecting differences in the network architecture. A higher crosslinking density generally leads to an increased compressive modulus, which enhances the ability of the hydrogel to resist deformation under the applied loads. As shown in Figure 8, the compressive stress for both types of alginate–PPy (or alginate@PPy-NP) was 80 kPa, with a permanent deformation setting at different times. Calcium alginate@PPy-NP resulted in a more elastic composite hydrogel than the composite calcium alginate–polypyrrole. These results suggest potential nanoparticle-mediated reinforcement of the hydrogels, consistent with prior reports of enhanced compressive performance in nanocomposite hydrogels [49,50]. Conversely, the calcium alginate–PPy system (Figure 8b) showed moderate improvements in mechanical integrity, with a single symmetric stress–strain peak that shifted slightly with increasing pyrrole content. Calcium alginate–PPy exhibited a sharp deformation pattern over a relatively short time (less than 100 s), whereas alginate@PPy-NP exhibited curve-like deformation behavior beyond 180 s. This is similar to the report by Wright et al. [24], who reported that in situ-generated polypyrrole resulted in a brittle hydrogel film at certain concentrations.

Conductive hydrogels can be engineered by incorporating conductive materials such as conductive polymers or nanoparticles into the hydrogel matrix. In this study, we demonstrated the possibility of improving the electrical conductivity of hydrogels through covalent the addition of a conductive polypyrrole polymer. To achieve a conductive hydrogel matrix, pyrrole monomers were added alongside pyrrole-functionalized sodium alginate to ensure a sufficient concentration for in situ oxidative polymerization. At sub-threshold concentrations, pyrrole tends to form short oligomers that lack the extended π-conjugation necessary for electrical conductivity [51]. The system facilitated the development of a conductive network throughout the hydrogel by reaching the critical concentration required for polypyrrole chain formation. The presence of covalently grafted pyrrole units promotes the localized polymerization and anchoring of polypyrrole within the alginate matrix. Furthermore, the addition of chemically synthesized polypyrrole nanoparticles (PPy-NPs) provided an additional conductive phase and contributed to mechanical reinforcement. This dual-modification strategy was intentionally employed to combine a uniform network integration with enhanced conductivity and mechanical resilience.

Electrical characterization showed that the integration of PPy imparted conductivity to the hydrogels, with a clear trend of increasing conductivity with increasing PPy content (Figure 9). Different oxidants, such as hydrogen peroxide, ferric chloride, and ammonium persulfate (APS), have been used to synthesize polypyrrole from its monomer precursor. Ferric chloride and APS exhibited fast reaction rates (Supplementary Figure S4). Because ferric ions, as multivalent cations, can interfere with sodium alginate crosslinking, APS was selected as the preferred oxidant to prepare polypyrrole [52] because of its compatibility with calcium ion-based alginate crosslinking, avoiding interference from Fe^3^⁺ ions. Polypyrrole-incorporated calcium–alginate hydrogels exhibited electrical conductivity that increased proportionally with polypyrrole content. The conductivity values obtained (Figure 9) are within the range previously reported for conductive polysaccharide-based hydrogels. For example, Guo et al. [53] reported that the electrical conductivity of pure chitosan (∼3.13 × 10^−8^ S/cm) increased to 2.97 × 10^−5^ S/cm upon the incorporation of 10% aniline as an intrinsically conducting component. In our system, crosslinked calcium alginate gels conducted electricity primarily through mobile ion transport, which was further enhanced by the introduction of a polypyrrole backbone featuring conjugated π-electron systems. This synergy results in a tunable and enhanced conductive profile, supporting the suitability of the hydrogel for electrically responsive tissue-engineering applications.

Our findings align with those of previous studies, demonstrating the role of conductive polymers in enhancing the functional performance of hydrogel networks. Wright et al. [24] reported that the in situ polymerization of polypyrrole within calcium alginate yielded brittle films with limited mechanical resilience. By contrast, our nanoparticle-blended approach (alginate@PPy-NP) achieved significantly improved elasticity, mechanical robustness, and compressive stability. Furthermore, the conductivity values observed in our composite system, particularly at higher polypyrrole concentrations, are comparable to or even exceed those reported for similar systems. For instance, hyaluronic acid hydrogels modified with polypyrrole have demonstrated electrical conductivities of approximately 7.3 mS/cm [54], highlighting the effectiveness of our dual-modification strategy. Collectively, these comparisons underscore the versatility of our approach for engineering electroconductive hydrogels with customizable electrical and mechanical properties. Scanning electron microscopy (SEM) confirmed the nanoscale morphology (0.29182 ± 0.06076 µm), revealing uniform spherical particles (Figure 5a) with rough surfaces, stable integration within the alginate matrix, and elemental distribution (Figure 5b). The influence of these nanoparticles is evident in extrusion-based 3D printing and enhanced mechanical properties. The calcium alginate@PPy-NP constructs exhibited concentration-dependent improvement in print fidelity. At 1% PPy-NP, the constructs showed relatively well-defined pores and improved grid uniformity compared to lower concentrations (Figure 13), although some filament spreading and edge blurring were still observed, particularly at the filament intersections. In contrast, constructs printed with 0.33% and 0.17% PPy-NPs doping displayed poor resolution, with visible pore collapse and filament merging, emphasizing the importance of nanoparticle concentration in maintaining structural stability. Printability was further enhanced by primary ionic crosslinking with 10 mM Ca^2^⁺, which increased ink viscosity and improved extrusion stability. Additionally, printing within a support bath preserved the grid geometry even at higher speeds, demonstrating the robustness of the bioink formulation under varying extrusion conditions and its suitability for diverse tissue engineering applications.

To assess the planar print precision, the printing factor (Pf) was calculated using ImageJ by comparing the measured pore area with the designed geometry. This semi-quantitative metric, adapted from previously established methods, reflects the roundness and uniformity of printed filaments. A Pf value closer to 1 indicates better pore shape fidelity [28,29]. Although Pf provides valuable insight into in-plane accuracy, it does not directly assess the vertical resolution or interlayer adhesion. Although not quantitatively evaluated in this study, consistent filament stacking and minimal delamination were observed, particularly for the 1% PPy-NP constructs. These observations suggest good layer-to-layer cohesion. Future work will incorporate z-resolution analysis and mechanical testing of interlayer adhesion to fully characterize the 3D print fidelity and mechanical integration of multilayered constructs.

Taken together, the integration of polypyrrole into calcium alginate via covalent polymerization or nanoparticle dispersion significantly enhanced the physicochemical and functional properties of the resulting electrically conductive hydrogels. These modifications improve rheological behavior, mechanical integrity, electrical conductivity, and print fidelity, all of which are critical parameters for bioprinting applications. The ability to tailor these properties through pyrrole concentration and formulation methods makes these bioinks promising candidates for extrusion-based tissue engineering, particularly in scenarios requiring both mechanical robustness and electrical activity, such as neural or bone tissue regeneration.

## 5. Conclusions

This study aimed to develop and characterize electrically conductive alginate-based hydrogels by covalently integrating polypyrrole through EDC/NHS chemistry and incorporating chemically synthesized polypyrrole nanoparticles, with the goal of enhancing their suitability as bioinks for 3D bioprinting and tissue engineering applications. The resultant alginate–polypyrrole (alginate–PPy) and alginate@PPy-NP hydrogels exhibited tunable viscoelasticity, improved compressive strength, and enhanced electrical conductivity, all of which are critical for maintaining shape fidelity during extrusion and for supporting cell-laden constructs.

Among the two systems, calcium alginate@PPy-NP provided greater mechanical robustness and print resolution, whereas calcium alginate–PPy offered a more uniform gel matrix appropriate for applications requiring soft, cell-encapsulating hydrogels. Both systems demonstrated shear-thinning behavior and maintained printable characteristics under various extrusion conditions.

However, this study has certain limitations. The long-term stability and biocompatibility of the conductive hydrogels under physiological conditions were not assessed in this study and warrant further investigation. Additionally, the influence of electrical conductivity on specific cellular responses and tissue regeneration outcomes remains to be explored in depth.

Future research should focus on the in vitro and in vivo biological performances of these conductive hydrogels, including cell viability, proliferation, and differentiation. Expanding the system to include stimuli-responsive or drug-releasing functionalities could further enhance its utility in regenerative medicine, especially for electrically active tissues, such as nerves and cardiac muscles.

## Figures and Tables

**Table 1:** Bioink solution formulation.

Sample	Materials	[Stock]	[Final]
A	Alginate	2.5%	2%
	Ca^2+^	0.1 M	0.01 M
	Pyrrole	14.125 M	0.1 M
	DDW		
B	Alginate–pyrrole	2.5%	2.0%
	Alginate	2.5%	2%
	Ca^2+^	0.1 M	0.01 M
	Pyrrole	14.125 M	0.1 M
	DDW		

DDW is double-distilled water.

**Table 2:** High-resolution XPS peak assignments and atomic percentages for sodium alginate and the alginate–pyrrole.

Element	Scan Assignment	Binding Energy (eV)	Atomic % (Alginate)	Atomic % (Alginate–Pyrrole)	Assignment Description
C1s	Scan A	283.2	3.96	8.27	Aliphatic C-C/C-H
C1s	Scan B	284.89	22.29	12.94	C-O, C-N
C1s	Scan C	286.49	20.23	13.62	C-O/C-N (ether or amine)
C1s	Scan D	288.08/288.4	7.1	33.63	O-C=O (carboxyl)/-CONH- (amide)
N1s	Scan A	-	-	4.43	Pyrrole ring nitrogen (C-N=C)
N1s	Scan B	-	-	3.67	Amide nitrogen (-NH-C=O)
O1s	Scan A	529.76/531.15	5.12	10.02	O=C (carbonyl oxygen)
O1s	Scan B	531.48/533.15	27.79	8.52	C-OH/C-O-C
O1s	Scan C	533.18/536.56	13.5	4.9	Possible oxidized or shifted O state

Note: “-” indicates no detectable peak in the unmodified alginate sample. Peak labels (A–D) correspond to deconvoluted sub-peaks within each elemental scan. The significant increase in N 1s content and the shift and intensification of C1s and O1s peaks in the alginate–pyrrole show amide bond formation.

**Table 3:** Summary of frequency sweep rheological parameters for calcium alginate-based inks.

Material	PPy Concentration	G′ @ 1 Hz (Pa)	G″ @ 1 Hz (Pa)	|η*| (Pa·s) @ 1 Hz	tan δ (G″/G′)	Interpretation	G′ > G″?
Calcium alginate		28.64	17.04	5.304	0.595	Moderate elasticity	Yes
Alginate/pyrrole	0.05 M	24.77	23.72	4.745	0.958	Low elasticity	Yes
Alginate/pyrrole	0.1 M	39.22	18.75	6.918	0.478	Improved elasticity	Yes
Alginate–pyrrole+alginate/pyrrole	0.1 M	49.9	24.92	8.876	0.499	Good elasticity	Yes
Alginate–PPy-NP	0.17%	49.7	19.74	8.511	0.397	Good elasticity	Yes
Alginate–PPy-NP	0.3%	100.8	24.91	16.53	0.247	High elasticity	Yes
Alginate–PPy-NP	1%	1770.0	119.5	282.3	0.068	Highest elasticity	Yes

**Table 4:** Steady-state flow behavior of calcium alginate-based inks.

Material	Concentration (% *w*/*v*)	η @ 1 s^−1^ (Pa·s)	η @ 100 s^−1^ (Pa·s)	Yield Stress (Pa)	Shear-Thinning Index (n)	Flow Behavior
Calcium alginate	—	15.1	0.85	13.24	0.66	Shear-thinning
Alginate/PPy	0.05 M	12.3	0.803	12.4	0.68	Shear-thinning
	0.1 M	17.2	0.955	13.96	0.69	Shear-thinning
Alginate–pyrrole/PPy	0.1 M	22.72	1.054	14.87	0.7	Shear-thinning
Alginate@PPy-NP	0.17%	31.99	1.017	15.08	0.72	Shear-thinning
	0.33%	34.74	5.531	21.33	0.45	Moderate shear-thinning
	1.0%	159.5	1.06	23.66	0.73	Shear-thinning

**Table 5:** Condensed interpretation of rheology and printability.

Material	Concentration	Rheology Summary	Printability
Alginate	—	Moderate G′, G″; shear-thinning; tan δ ≈ 0.6	Acceptable
Alginate/pyrrole	0.05–0.1 M	G′ ≈ G″ at 0.05 M; increased G′ at 0.1 M; improved tan δ	Acceptable to improved
Alginate–PPy	0.1 M	Further ↑ G′, lower tan δ; more elastic	Improved
Alginate@PPy-NP	0.17–0.3%	High G′, moderate tan δ; increasing yield stress	High fidelity, stable
Alginate@PPy-NP	1.0%	Highest G′, highest yield stress; low tan δ	Best grid fidelity

The ↑ means that the parameter increases

## Data Availability

The original results presented in this study are included in this article and the Supplementary Materials. Further inquiries in terms of data can be found in our footprint records (https://footprints-b291f.web.app/, accessed on 23 April 2025). Authorization for access may be granted by the corresponding author (rsmarks@bgu.ac.il).

## Supplementary Materials

The following supporting information can be downloaded at https://www.mdpi.com/article/10.3390/ma18133120/s1. Figure S1: 13C NMR spectrum of the synthesized aminopropyl pyrrole; Figure S2: Mass Spectrum of Synthesized Aminopropyl Pyrrole; Figure S3: ATR-FTIR Spectra of Alginate-(amino)-pyrrole. Figure S4: Comparison of deconvoluted XPS spectrum peaks (a–d) and survey analysis (d) of lyophilized sodium alginate; Figure S5: Different oxidants for the synthesis of polypyrrole; Figure S6: Calibration curve of pyrrole monomer; Figure S7: Frequency sweep of alginate and alginate-pyrrole/pyrrole; Figure S8: Frequency sweep of alginate and alginate@PPy-NP; Figure S9: G′/G″ ratio of alginate, alginate-pyrrole and alginate/polypyrrole at frequencies of 1.0 and 10 Hz; Figure S10: Viscosity of alginate and alginate/pyrrole; Figure S11: Electrical conductivity of polypyrrole modified alginate; Figure S12: Dynamic light scattering (DLS) intensity distribution of polypyrrole nanoparticles (PPy-NPs) dispersed in 1-methylpyrrolidone (0.1 mg/mL); Figure S13 TEM micrographs of chemically synthesized polypyrrole nanoparticles (PPy-NPs) at 100,000× magnification; Figure S14: TGA profiles of alginate-pyrrole; Figure S15: TGA profile of sodium alginate; Table S1: Functional groups identified in the synthesized aminopropyl pyrrole and alginate-pyrrole; Peak Table S2: (a) XPS data for Alginate, (b): XPS Peak Fit Table of Alginate; Table S3: (a) XPS Peak Fit Table of Alginate, (b) Alginate-pyrrole Peak Fit; Table S4: Printing Parameters optimization; Table S5: Bioink Formulation.

## Abbreviations

The following abbreviations are used in this manuscript:

DDW	Double-distilled water
DMEM	Dulbecco’s Modified Eagle Medium
EDC	1-Ethyl-3-[3-dimethylaminopropyl] carbodiimide hydrochloride
FRESH	Freeform Reversible Embedding of Suspended Hydrogels
NHS	N-hydroxysuccinimide
NMR	Nuclear magnetic resonance
PPy-NP	Polypyrrole nanoparticles
SEM	Scanning Electron Microscope
XPS	X-ray photoelectron spectroscopy
