# Supplementary material for: Development of Covalently Functionalized Alginate–Pyrrole and Polypyrrole–Alginate Nanocomposites as 3D Printable Electroconductive Bioinks
Source: Materials (Basel). 2025 Jul 1;18(13):3120. doi: 10.3390/ma18133120 (PMC12251084; doi:10.3390/ma18133120)
Supplement: Supplementary file 1 [file materials-18-03120-s001.zip › materials-3669791-supplementary.pdf]

Supplementary Materials

Protocol S1: Preparation of Gelatin Slurry Support Bath

According to the protocol of Hinton et al. [1]. A gelatin slurry support bath was prepared by dissolving 4.5% (w/v) gelatin (Type A, Thermo Fisher Scientific) in 11 mM $CaCl_2$ (Sigma-Aldrich), followed by gelation at 4 °C for 12 hours in a 500-mL container. After gelation, 350 mL of chilled 11 mM $CaCl_2$ was added, and the mixture was blended at pulse speed for 30 to 120 seconds using a consumer-grade blender. The resulting slurry was transferred into 50-mL conical tubes and centrifuged at 4200 rpm for 2 minutes to sediment the gelatin particles. The supernatant was discarded and replaced with fresh cold 11 mM $CaCl_2$. The slurry was then vortexed to resuspend the particles and centrifuged again. This washing process was repeated until the supernatant was free of bubbles, indicating the effective removal of soluble gelatin. The final gelatin slurry was stored at 4 °C until use. Before printing, the slurry was transferred to a suitable container, and any excess liquid was carefully removed using lint-free wipes, yielding a support material with Bingham plastic-like behavior. All 3D printing experiments were performed using gelatin slurries blended for 120 seconds.

1.0 NMR Spectroscopic studies

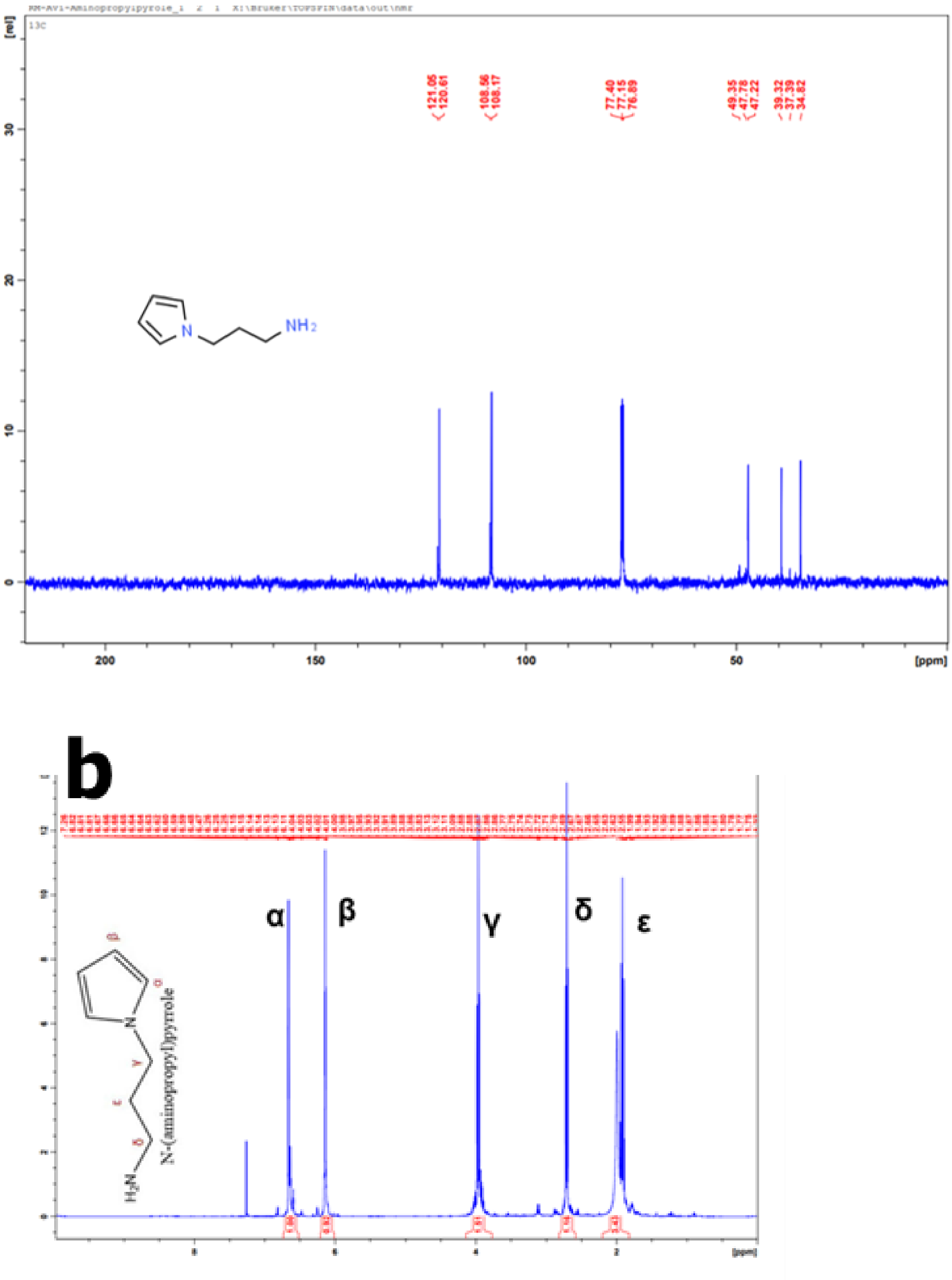

Figure S1: ^{13}C NMR spectrum of the synthesized aminopropyl pyrrole

^{13}C NMR of amines shows that carbon attached to nitrogen appears in 10-65 ppm region, shifted slightly downfield than alkane carbons because of the de-shielding caused by the electron withdrawing effect of nitrogen.

Mass Spectrum of Synthesized Aminopropyl Pyrrole

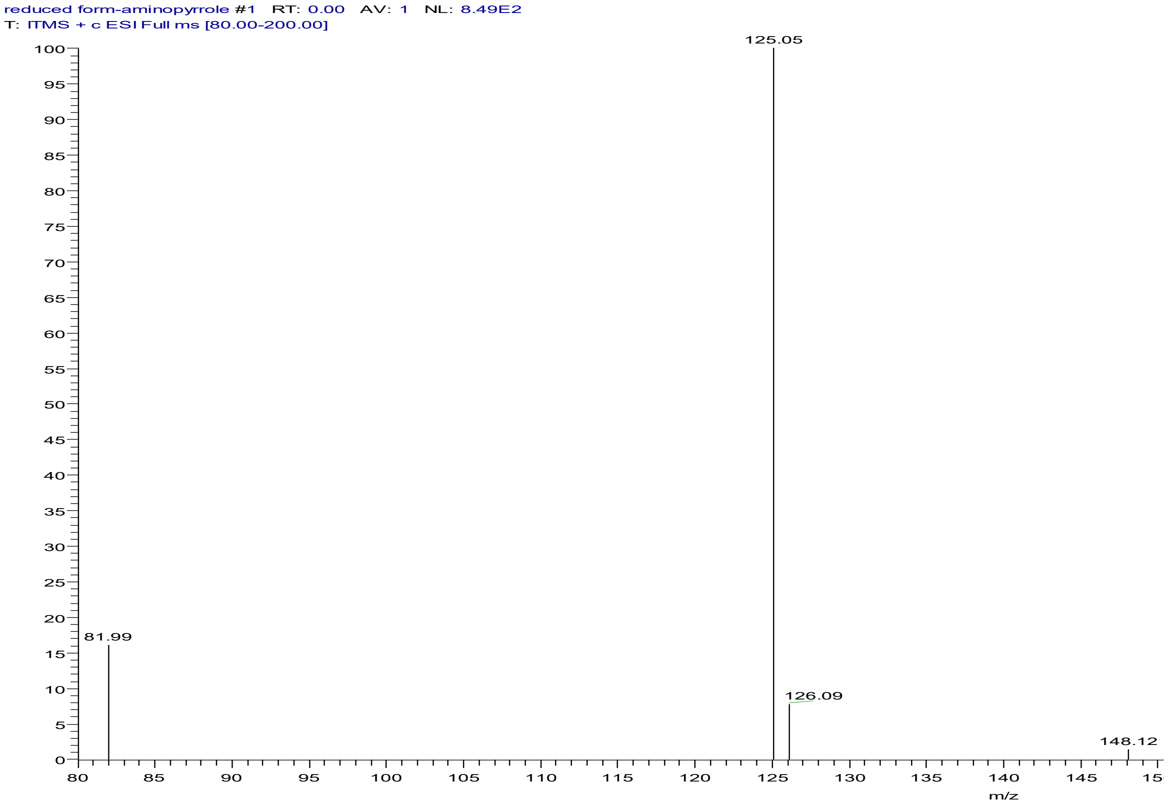

Figure S2: Mass Spectrum of Synthesized Aminopropyl Pyrrole

FTIR spectroscopy of alginate-pyrrole

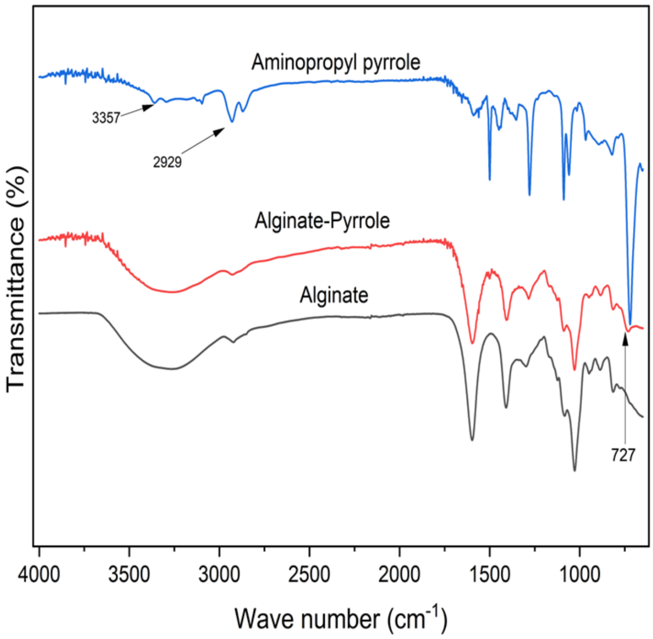

Figure S3: ATR-FTIR Spectra of Alginate-(amino)-pyrrole.

The bands corresponding to the different functional groups are summarized on the table below:

Table S1: Functional groups identified in the synthesized aminopropyl pyrrole and alginate-pyrrole

Determination of suitable oxidants

**Functional group/Change in vibration**	**Wave number (cm^{-1})**
C-H wag of pyrrole	729
Symmetric and asymmetric NH_2 stretch	3325, 2929
C=0 stretching amide	1650
C-N stretch	1278
NH_2 Scissoring	729
NH_2 wagging	1627
In-plane NH bending	1567
Out-plane NH bending	720

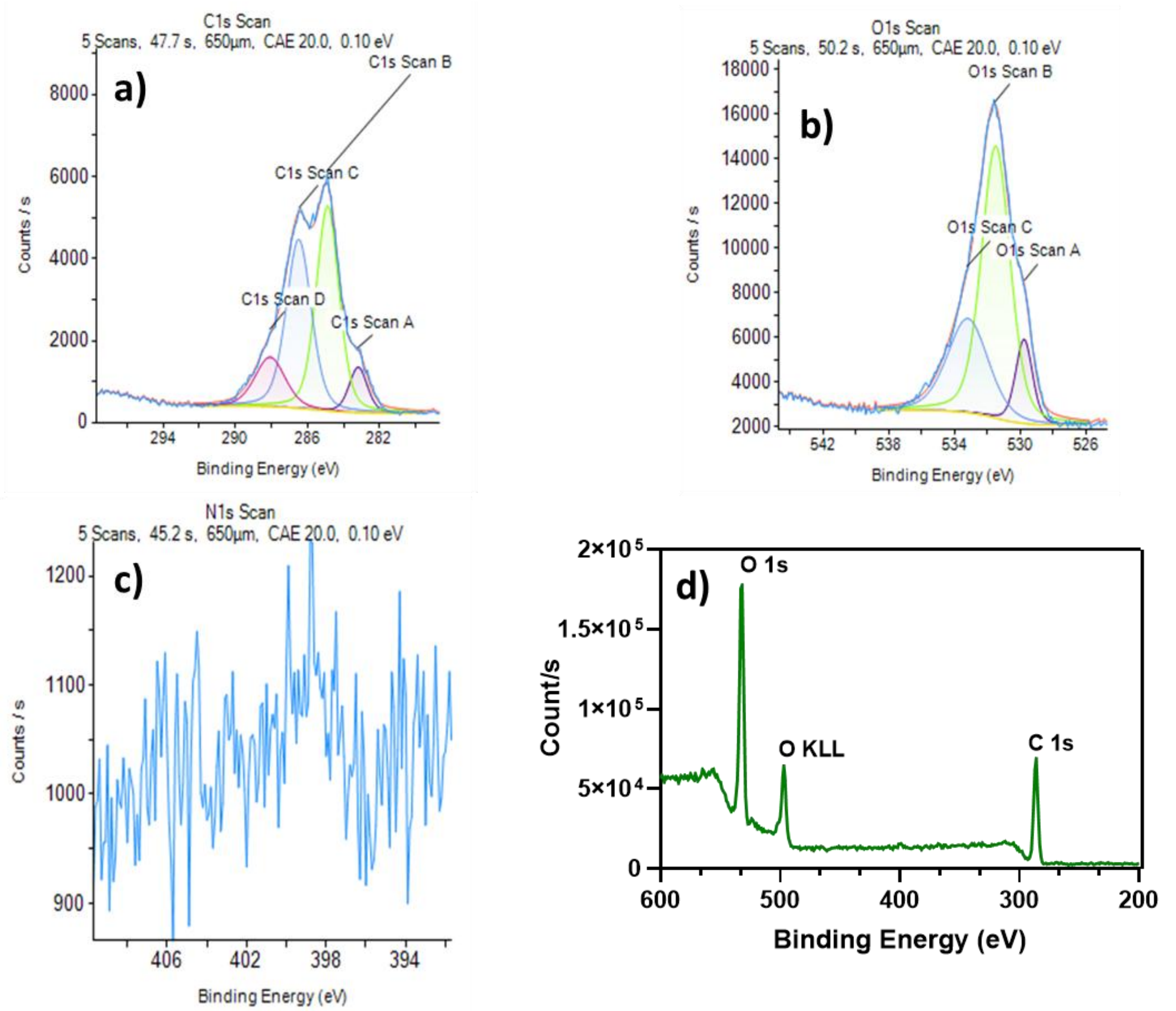

Figure S4: Comparison of deconvoluted XPS spectrum peaks (a-d) and survey analysis (d) of lyophilized sodium alginate. The deconvoluted peaks were assigned to chemical groups based on the binding energies of the peaks (N1s, O1s, and C1s).

Peak Table S2a : Alginate

**Alginate Peak Table S2a :**										
Name	Start BE	Peak BE	End BE	Height CPS	FWHM eV	Area (P) CPS.eV	Area (N) TPP-2M	Atomic %	Peak Type	Q
C1s Scan A	292.28	283.2	279.52	1113.1	1.19	1576.83	0.04	3.96	Fitted	1
C1s Scan B	292.28	284.89	279.52	5004.61	1.49	8869.74	0.2	22.29	Fitted	1
C1s Scan C	292.28	286.49	279.52	4122.16	1.65	8045.06	0.18	20.23	Fitted	1
C1s Scan D	292.28	288.08	279.52	1209.35	1.98	2822.43	0.06	7.1	Fitted	1
O1s Scan A	538.6	529.76	525.81	3775.72	1.22	5496.58	0.05	5.12	Fitted	1
O1s Scan B	538.6	531.48	525.81	12196.05	2.08	29803.11	0.25	27.79	Fitted	1
O1s Scan C	538.6	533.18	525.81	4219.62	2.95	14468.49	0.12	13.5	Fitted	1

Alginate

Peak Fit Table S2b:

**C1s Scan**											
Ref.	Name	Peak BE	Height CPS	Height Ratio	Area CPS.eV	Area Ratio	FWHM fit param (eV)	L/G Mix (%) Sum	Tail Mix (%)	Tail Height (%)	Tail Exponent
H	C1s Scan A	283.2	1113.1	0.22	1576.83	0.18	1.19	30	100	0	0
							0.5 : 3.5	fixed	fixed	fixed	fixed
I	C1s Scan B	284.89	5004.61	1	8869.74	1	1.49	30	100	0	0
							0.5 : 3.5	fixed	fixed	fixed	fixed
J	C1s Scan C	286.49	4122.16	0.82	8045.06	0.91	1.64	30	100	0	0
							0.5 : 3.5	fixed	fixed	fixed	fixed
	C1s Scan D	288.08	1209.35	0.24	2822.43	0.32	1.97	30	100	0	0
**O1s Scan**											
Ref.	Name	Peak BE	Height CPS	Height Ratio	Area CPS.eV	Area Ratio	FWHM fit param (eV)	L/G Mix (%) Sum	Tail Mix (%)	Tail Height (%)	Tail Exponent
L	O1s Scan A	529.76	3775.72	0.31	5496.58	0.18	1.22	30	100	0	0
							0.5 : 3.5	fixed	fixed	fixed	fixed
M	O1s Scan B	531.48	12196.05	1	29803.11	1	2.07	30	100	0	0
							0.5 : 3.5	fixed	fixed	fixed	fixed
N	O1s Scan C	533.18	4219.62	0.35	14468.49	0.49	2.93	30	100	0	0
							0.5 : 3.5	fixed	fixed	fixed	fixed

Peak Table S3a : Alginate-Pyrrole

Name	Start BE	Peak BE	End BE	Height CPS	FWHM eV	Area (P) CPS.eV	Area (N) TPP-2M	Atomic %	Peak Type	Q
C1s Scan A	293.25	283.26	279.58	1490.5	1.3	2299.97	0.05	8.27	Fitted	1
C1s Scan B	293.25	284.74	279.58	1961.29	1.55	3600.31	0.08	12.94	Fitted	1
C1s Scan C	293.25	286.33	279.58	1711.06	1.88	3788.41	0.09	13.62	Fitted	1
C1s Scan D	293.25	288.4	279.58	2613	3.08	9347.19	0.21	33.63	Fitted	1
N1s Scan A	407.91	399.5	393.5	495.15	3.54	2006.06	0.03	4.43	Fitted	1
N1s Scan B	407.91	402.8	393.5	411.02	3.53	1664.23	0.02	3.67	Fitted	1
O1s Scan A	541.07	531.15	525.1	2426.36	2.66	7511.07	0.06	10.02	Fitted	1
O1s Scan B	541.07	533.15	525.1	1727.39	3.19	6383.44	0.05	8.52	Fitted	1
O1s Scan C	541.07	536.56	525.1	983.82	3.21	3667.84	0.03	4.9	Fitted	1

Table S3b: Alginate-pyrrole Peak Fit

**C1s Scan**											
Ref.	Name	Peak BE	Height CPS	Height Ratio	Area CPS.eV	Area Ratio	FWHM fit param (eV)	L/G Mix (%) Sum	Tail Mix (%)	Tail Height (%)	Tail Exponent
J	C1s Scan A	283.26	1490.5	0.57	2299.97	0.25	1.3	30	100	0	0
							0.5 : 3.5	fixed	fixed	fixed	fixed
K	C1s Scan B	284.74	1961.29	0.75	3600.31	0.39	1.55	30	100	0	0
							0.5 : 3.5	fixed	fixed	fixed	fixed
L	C1s Scan C	286.33	1711.06	0.65	3788.41	0.41	1.87	30	100	0	0
							0.5 : 3.5	fixed	fixed	fixed	fixed
	C1s Scan D	288.4	2613	1	9347.19	1	3.06	30	100	0	0
**N1s Scan**											
Ref.	Name	Peak BE	Height CPS	Height Ratio	Area CPS.eV	Area Ratio	FWHM fit param (eV)	L/G Mix (%) Sum	Tail Mix (%)	Tail Height (%)	Tail Exponent
N	N1s Scan A	399.5	495.15	1	2006.06	1	3.5	30	100	0	0
							0.5 : 3.5	fixed	fixed	fixed	fixed
O	N1s Scan B	402.8	411.02	0.83	1664.23	0.83	3.5	30	100	0	0
							0.5 : 3.5	fixed	fixed	fixed	fixed
**O1s Scan**											
Ref.	Name	Peak BE	Height CPS	Height Ratio	Area CPS.eV	Area Ratio	FWHM fit param (eV)	L/G Mix (%) Sum	Tail Mix (%)	Tail Height (%)	Tail Exponent
P	O1s Scan A	531.15	2426.36	1	7511.07	1	2.64	30	100	0	0
							0.5 : 3.5	fixed	fixed	fixed	fixed
Q	O1s Scan B	533.15	1727.39	0.71	6383.44	0.85	3.16	30	100	0	0
							0.5 : 3.5	fixed	fixed	fixed	fixed
R	O1s Scan C	536.56	983.82	0.41	3667.84	0.49	3.19	30	100	0	0
							0.5 : 3.5	fixed	fixed	fixed	fixed

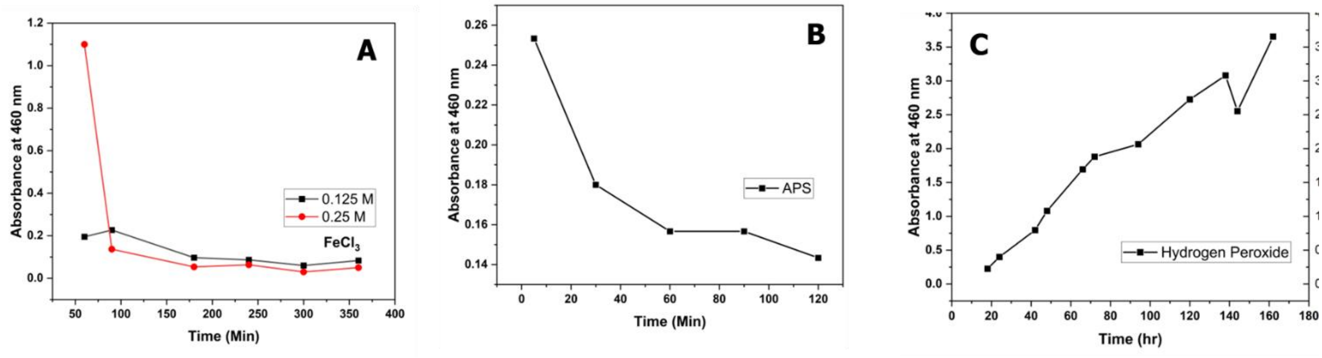

Figure S5: Different oxidants for the synthesis of polypyrrole. **(A.)** 0.25 and 0.125 M $FeCl_3.6H_20$ (molar ratio of 2.3:1, $FeCl_3.6H_20$: Pyrrole). **(B)** 0.15M Ammonium Persulfate (ratio, 2:3) **(C)** 0.2 M hydrogen peroxide (ratio 1:1.5)

UV-Vis quantification of the alginate-pyrrole conjugate

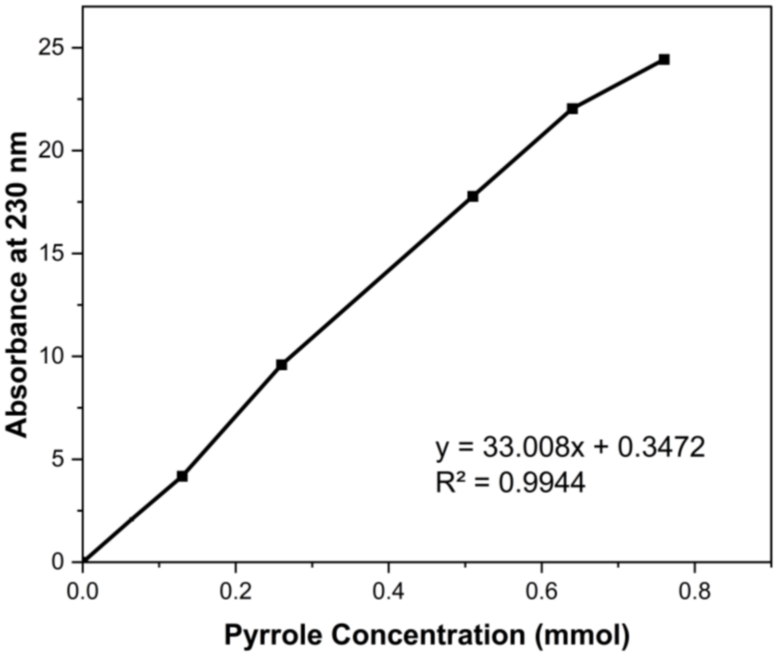

Figure S6: Calibration curve of pyrrole monomer

Rheological behavior of alginate, alginate-pyrrole and alginate/pyrrole

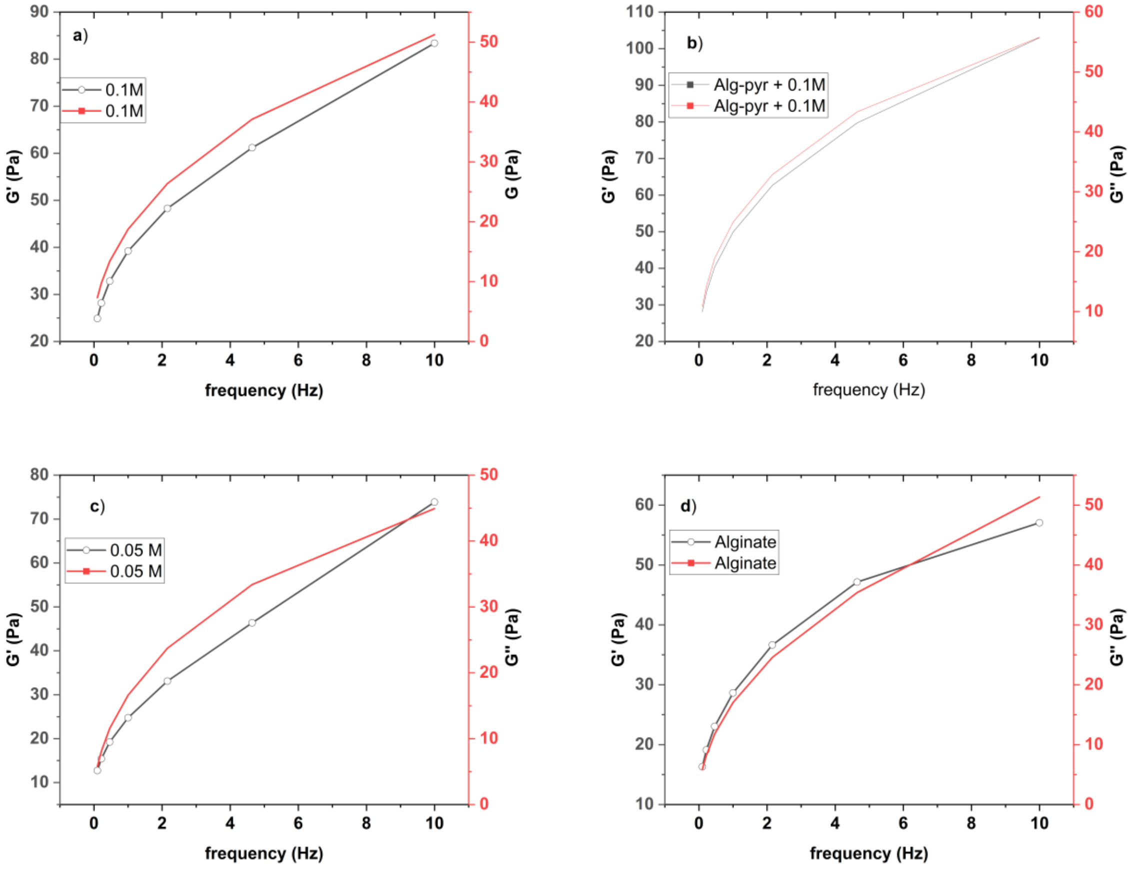

Figure S7: Frequency sweep of alginate and alginate-pyrrole/pyrrole

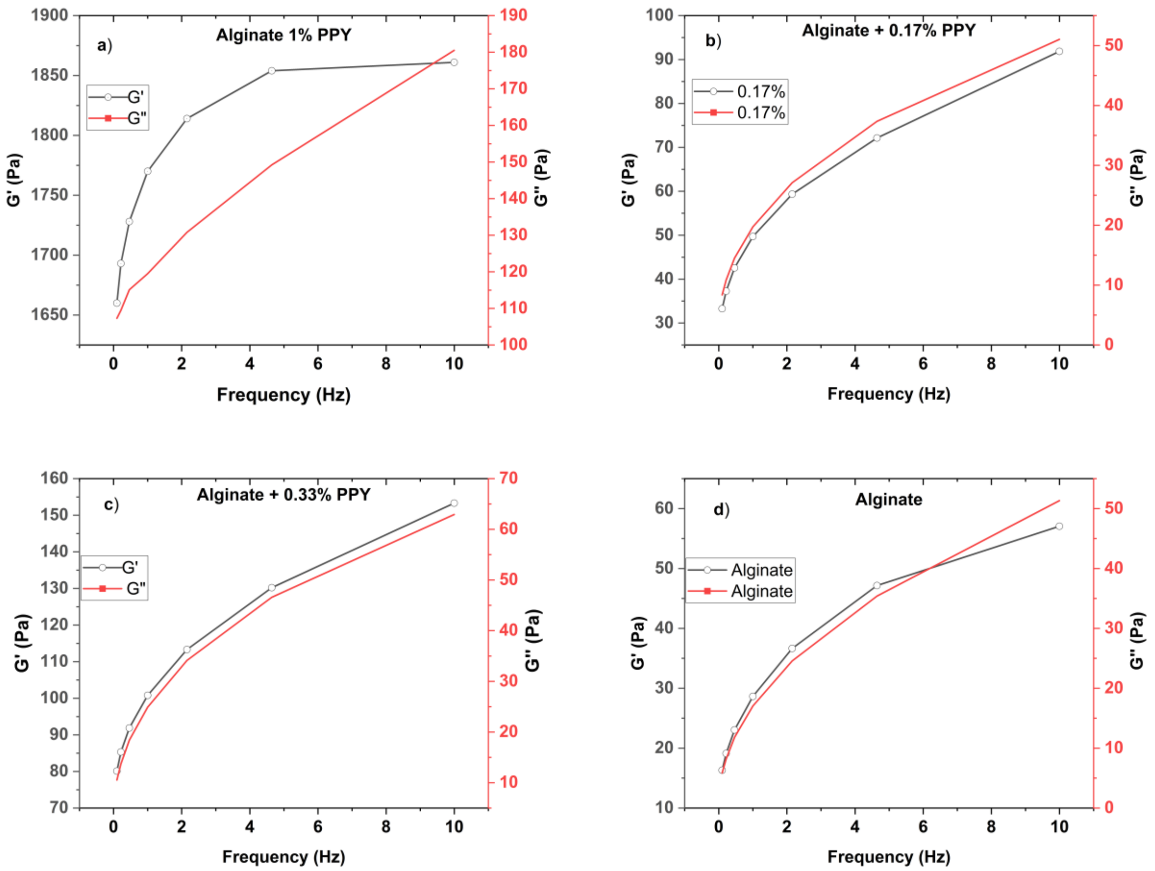

Figure S8: Frequency sweep of alginate and alginate@PPy-NP

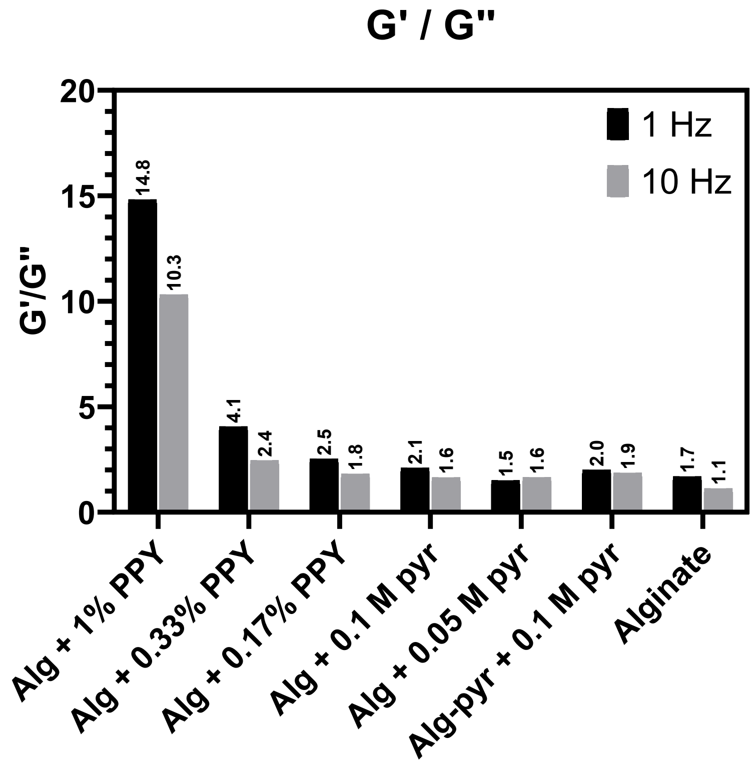

Figure S9: G'/G'' ratio of alginate, alginate-pyrrole and alginate/polypyrrole at frequencies of 1.0 and 10 Hz.

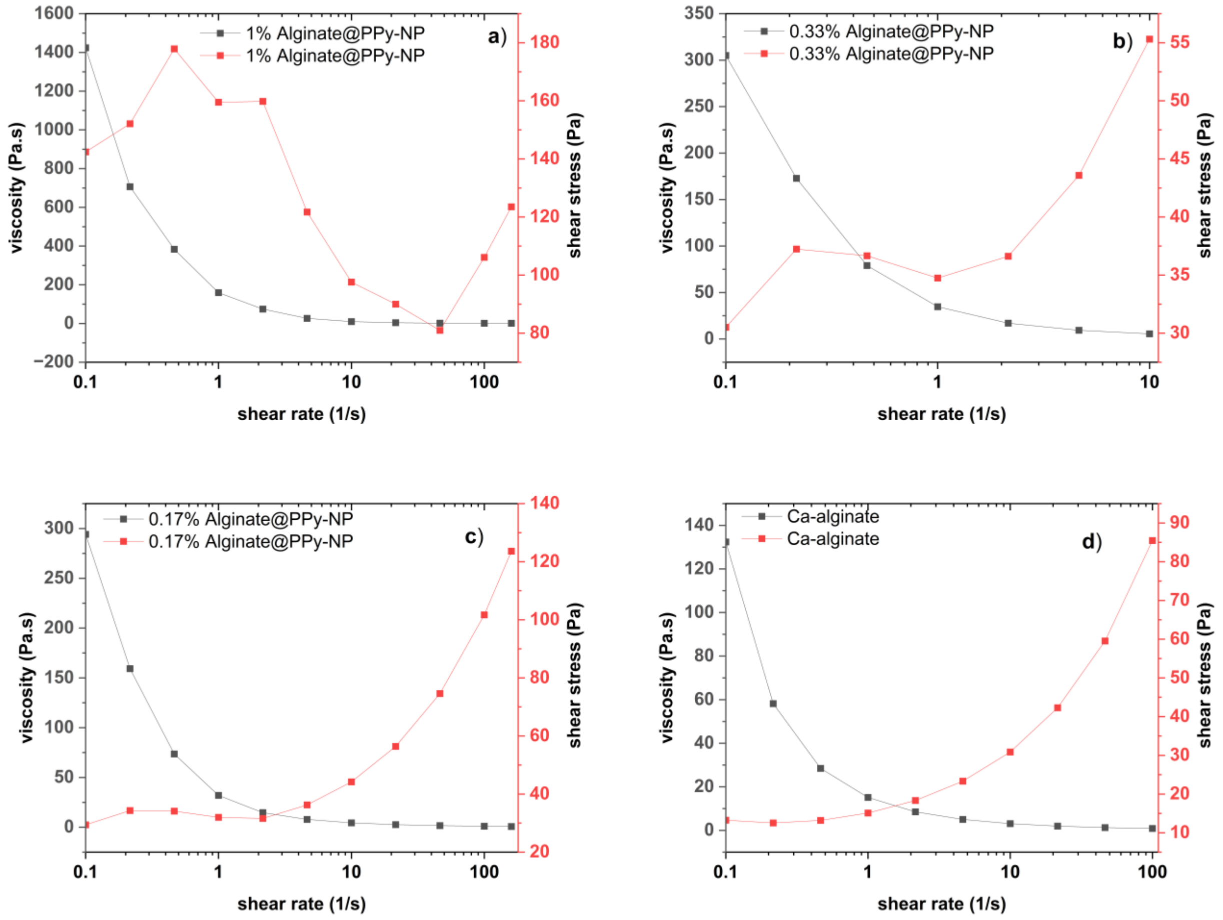

Figure S10: Viscosity of alginate and alginate/pyrrole

Electrical Properties

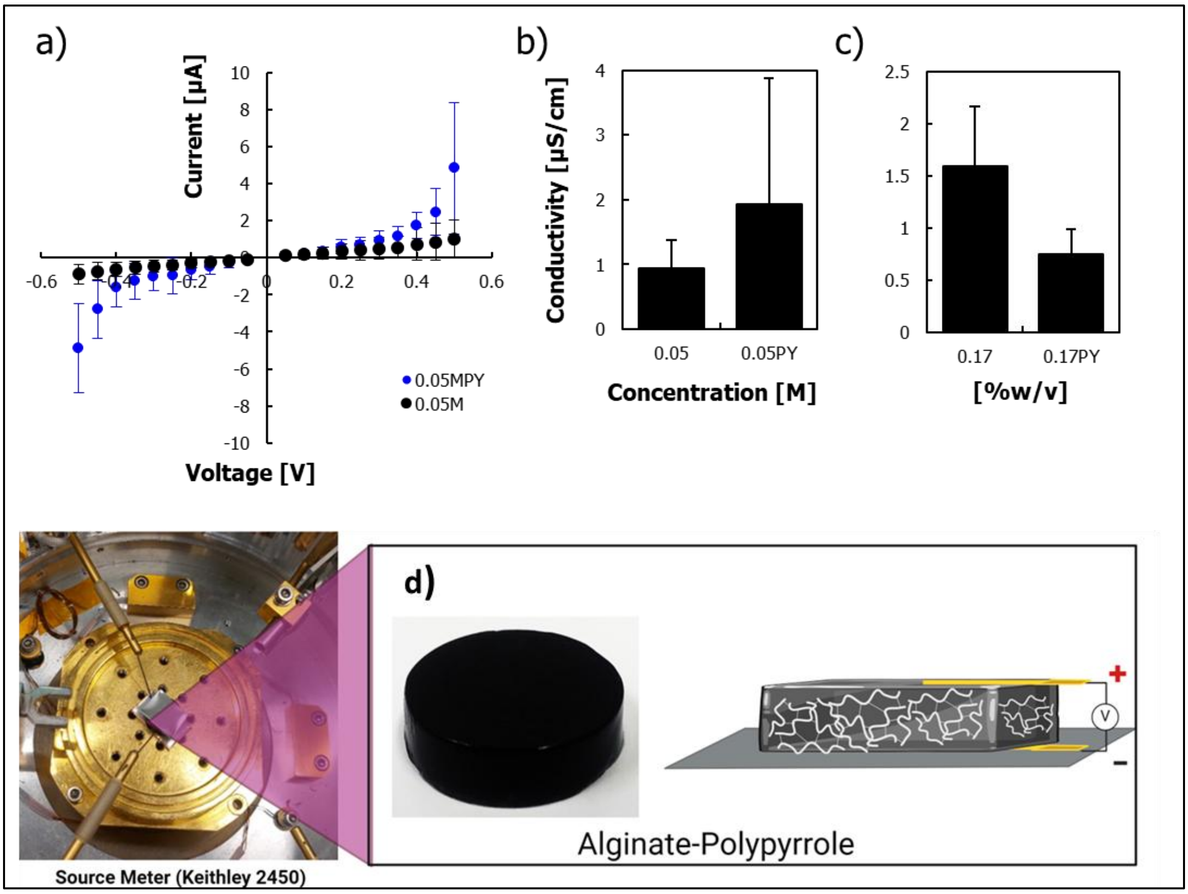

Figure S11: Electrical conductivity of polypyrrole modified alginate. a) The current voltage plot of alginate-pyrrole conjugates mixed with pyrrole monomer (expressed in molar, M) polypyrrole was generated in situ. 0.0 5 M means alginate mixed with 0.0 5 M pyrrole, while 0.05 Py means alginate-pyrrole mixed with 0.0 5 M pyrrole monomer. b) conductivity of alginate and alginate-pyrrole mixed with 0.05 M pyrrole followed by polymerization into polypyrrole (alginate-PPy). c) conductivity of alginate and alginate-pyrrole mixed with 0.17% w/v polypyrrole (alginate@PPy-NP). d) the schematic set-up used to acquire electrical conductivity.

Table S4 Printing Parameters optimization

Extrusion Parameters	Value
Nozzle diameter	250 μm
Strand thickness	200 μm
Height	0.8 mm
Strand spacing	2.25 mm
Speed	10-22 mm/s
Pressure	0.6-0.8 bar
Layers	4
Change in direction between layers	90°
Dimension	14 x 14 x 1

Table S5. Bioink Formulation

Sample	Materials	[Stock]	[Final]
**A**	Alginate	2.5%	2%
	Ca^{2+}	0.1 M	0.01 M
	Pyrrole	14.125 M	0.1 M
	DDW		
**B**	Alginate-pyrrole	2.5%	2.0%
	Alginate	2.5%	2%
	Ca^{2+}	0.1 M	0.01 M
	Pyrrole	14.125 M	0.1 M
	DDW		

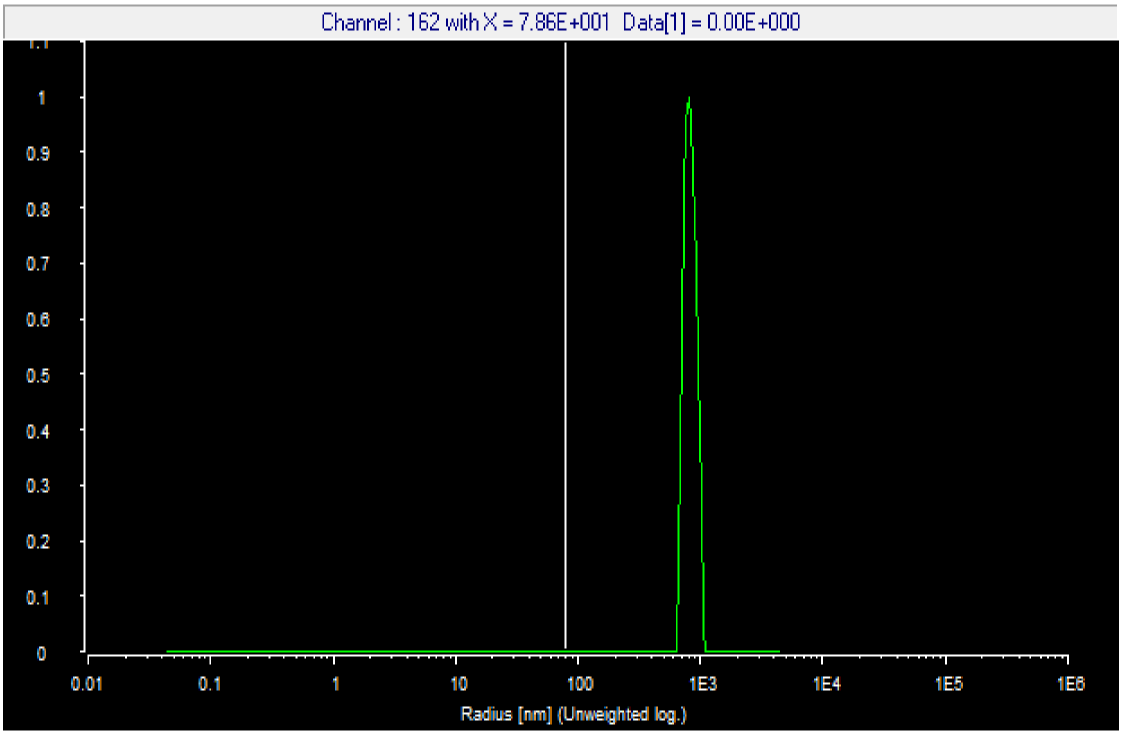

Figure S12: Dynamic light scattering (DLS) intensity distribution of polypyrrole nanoparticles (PPy-NPs) dispersed in 1-methylpyrrolidone (0.1 mg/mL), showing a narrow peak centered around 786 nm hydrodynamic radius, indicating moderate aggregation.

Transmission Electron Microscopy (TEM) Analysis of Polypyrrole Nanoparticles (PPy-NPs)

Transmission electron microscopy (TEM) was employed to further examine the morphology and size distribution of the synthesized polypyrrole nanoparticles (PPy-NPs). The TEM images reveal well-defined, nearly spherical particles exhibiting moderate aggregation. Representative measurements from multiple fields indicate particle diameters ranging from approximately 253 nm to 372 nm. The average particle diameter, based on the labeled measurements (372.07 nm, 294.01 nm, 265.87 nm, and 253.34 nm), is approximately 296 ± 50 nm. These dimensions are in agreement with SEM observations and complement DLS results, which reflected larger hydrodynamic radii due to solvation and aggregation in dispersion. Together, these data confirm the nanoscale nature of the synthesized PPy-NPs.

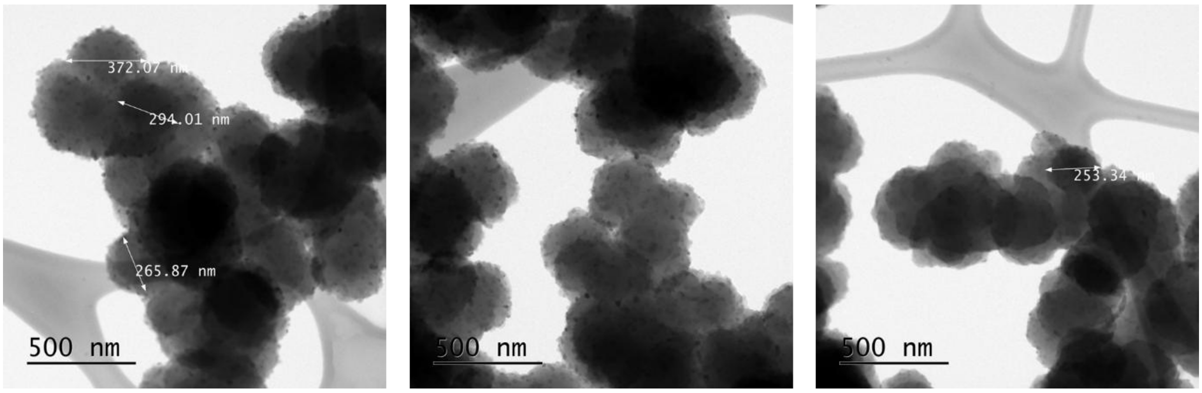

Figure 13: Supplementary Figure S12. TEM micrographs of chemically synthesized polypyrrole nanoparticles (PPy-NPs) at 100,000× magnification, showing individual spherical nanoparticles

with moderate aggregation. Scale bars represent 500 nm. Measured particle diameters indicate an average size of approximately 296 ± 50 nm.

2.5.4 Thermogravimetric Analysis (TGA)

Thermogravimetric analysis (TGA) was conducted to assess the thermal stability of sodium alginate (Na-alginate) and the synthesized alginate-pyrrole (alginate-PPy) composite. Approximately 2.2 mg of each dried sample was accurately weighed into an open alumina crucible and analyzed using TA Instruments Q500 and Q50 thermogravimetric analyzers (New Castle, Delaware, United States). The samples were heated from 50 °C to 1000 °C at a constant rate of 10 °C/min under a nitrogen atmosphere (flow rate: 60 mL/min). The resulting thermograms were used to compare the decomposition profiles and residual mass of the unmodified and functionalized alginate materials.

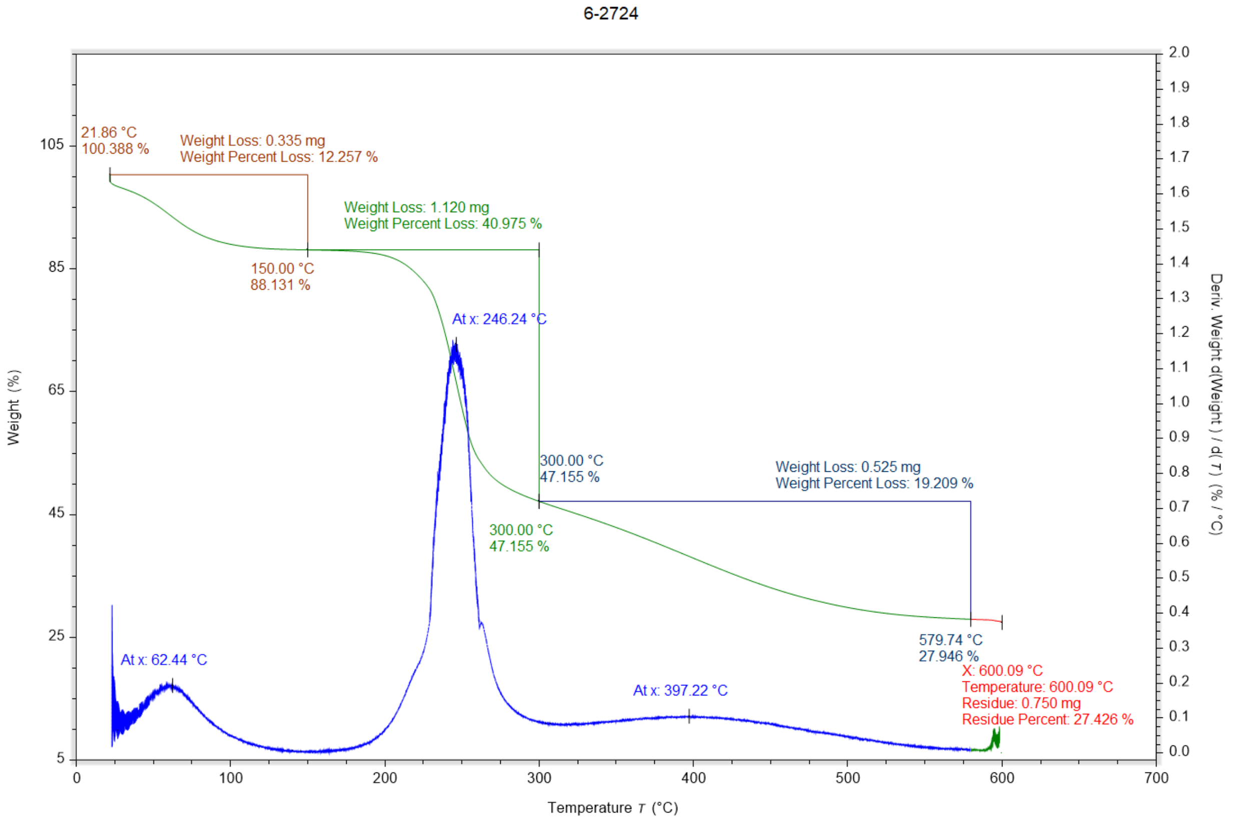

Figure S14: TGA profiles of alginate-pyrrole

Test	Custom
Test Name	TGA: Alginate-Pyrrole
Segment 1	Ramp 10.00 °C/min to 600.00 °C
Segment 2	Isothermal 8.0 min
Start Experiment After Weight Stabilization	No
Enable Air Cool	Yes
Air Cool Until Temperature Is Below	30.00 °C

End of Test Delay	0.00 minutes
Use default calibrations	Yes

Signal max

At x
62.44 °C
246.24 °C
397.22 °C

Weight change

Cursor points without text	Weight Loss	Weight Percent Loss
21.86 °C<br>100.388 %<br><br>150.00 °C<br>88.131 %	0.335 mg	12.257 %
150.00 °C<br>88.131 %<br><br>300.00 °C<br>47.155 %	1.120 mg	40.975 %
300.00 °C<br>47.155 %<br><br>579.74 °C<br>27.946 %	0.525 mg	19.209 %

Residue

X	Temperature	Residue	Residue Percent
600.09 °C	600.09 °C	0.750 mg	27.426 %

Alginate Alone

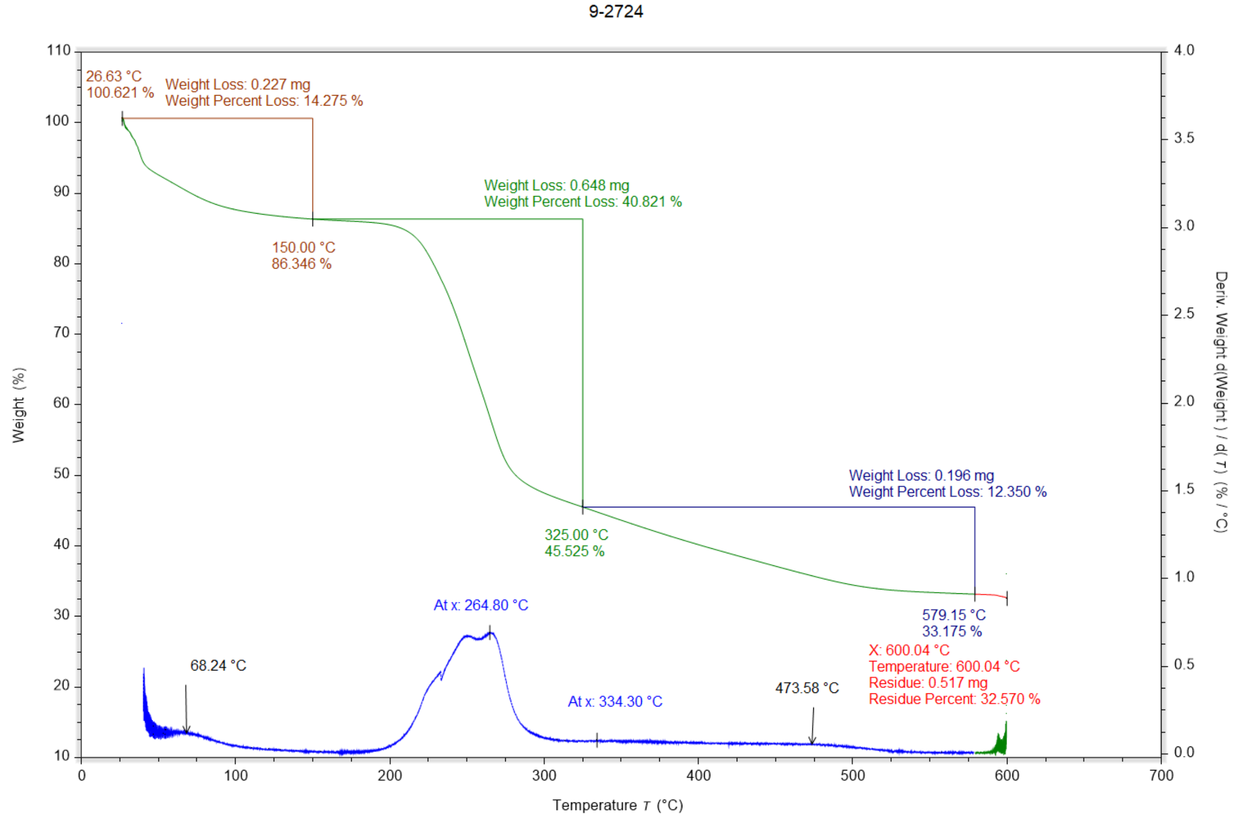

Figure S15: TGA profile of sodium alginate

Test	Custom
Test Name	TGA: Sodium Alginate
Segment 1	Ramp 10.00 °C/min to 600.00 °C
Segment 2	Isothermal 8.0 min
Start Experiment After Weight Stabilization	No
Enable Air Cool	Yes
Air Cool Until Temperature Is Below	30.00 °C
End of Test Delay	0.00 minutes
Use default calibrations	Yes

Weight change

Cursor points without text	Weight Loss	Weight Percent Loss
26.63 °C<br>100.621 %<br><br>150.00 °C<br>86.346 %	0.227 mg	14.275 %

Cursor points without text	Weight Loss	Weight Percent Loss
150.00 °C<br>86.346 %<br><br>325.00 °C<br>45.525 %	0.648 mg	40.821 %
325.00 °C<br>45.525 %<br><br>579.15 °C<br>33.175 %	0.196 mg	12.350 %

Residue

X	Temperature	Residue	Residue Percent
600.04 °C	600.04 °C	0.517 mg	32.570 %

Signal max

At x
264.80 °C
334.30 °C

1. Hinton, T.J.; Jallerat, Q.; Palchesko, R.N.; Park, J.H.; Grodzicki, M.S.; Shue, H.-J.; Ramadan, M.H.; Hudson, A.R.; Feinberg, A.W.J.S.a. Three-dimensional printing of complex biological structures by freeform reversible embedding of suspended hydrogels. **2015**, *1*, e1500758.
